# Activity outcomes after hip arthroplasty: an information tool based on patients’ experience captured in a hospital registry

**DOI:** 10.1186/s12891-025-09024-w

**Published:** 2025-08-20

**Authors:** S. Cole, G. Fabiano, C. Barea, S. Cullati, T. Agoritsas, N. Gutacker, A.J. Silman, D. Hannouche, A. Lübbeke, Rafael Pinedo-Villanueva

**Affiliations:** 1 Nuffield Department of Orthopaedics, Rheumatology and Musculoskeletal Sciences, University of Oxford, Oxford, UK; 2 Division of Orthopaedics & Trauma Surgery, Geneva University Hospitals and University of Geneva, Geneva, Switzerland; 3 Quality of Care Service, Department of Readaptation and Geriatrics, University Hospitals of Geneva, University of Geneva, Geneva, Switzerland; 4 Population Health Laboratory (#PopHealthLab), University of Fribourg, Fribourg, Switzerland; 5 Division General Internal Medicine, University Hospitals of Geneva, Geneva, Switzerland; 6 Department of Health Research Methods, Evidence, and Impact, McMaster University, Ontario, Canada; 7 Centre for Health Economics, University of York, York, UK

**Keywords:** Total hip arthroplasty, Patient reported outcome measures, Activity level outcomes, Information tool, Shared decision making

## Abstract

**Background and purpose:**

Patients receiving total hip arthroplasty (THA) have different expectations and concerns about their health outcomes after surgery. In this study we developed a tool based on registry data to inform patients and their clinicians about activity outcomes after THA.

**Methods:**

We used data from the Geneva Arthroplasty Registry (GAR) on patients receiving a primary elective THA between 1996 and 2019. The information tool was developed around five activity outcomes: getting in/out of the car, getting dressed autonomously, independence in weekly tasks, interference in social activities, and activity levels. Based on baseline predictors, conditional inference trees (CITs) were used to create clusters of patients with homogeneous activity outcomes at one, five and 10 years after surgery, rather than to predict individual probabilities.

**Results:**

In total, 14 CITs were generated based on 6,836 operations included in the tool. Overall, activity outcomes substantially improved at all three times points after surgery, with 1-year values mostly being the highest. While before surgery only about 10% of patients had none/slight limitations in activities of daily living, about 70% did one year after surgery. The SF12 mental component score (MCS), SF12 self-rated health (SRH), BMI, ASA score, and comorbidity count were the most recurring predictors of activity outcomes. Predictors and their relative importance changed at different time points for the same outcome. For example, for ability to get in/out the car, whilst clusters at year 1 were generated based on WOMAC function, SRH, mental health, WOMAC difficulty walking, and SF12 physical interference, at year 5, ASA score, BMI, SF12 physical & mental health, activity level, and socio-economic status were significant. Outcome profiles varied by clusters.

**Conclusion:**

Distinct activity outcomes clusters based on baseline patient characteristics were identified and knowing this can help inform patients’ expectation and meaningful discussions with clinicians about treatment decisions.

**Supplementary Information:**

The online version contains supplementary material available at 10.1186/s12891-025-09024-w.

## Introduction

Total hip arthroplasty (THA) is one of the most widely performed surgical procedures in orthopaedic practice. On average, OECD countries perform 182 hip replacement procedures for every 100,000 individuals. Switzerland ranks second with 307 procedures per 100,000 population [1].

In most patients, THA leads to improved clinical outcomes and activity capacity. However, some patients may achieve better outcomes than others and the effectiveness of the procedure is known to be moderated by pre-operative factors such as activity and radiological severity of OA [2]. Also, surgeons and patients might not be aligned on what to expect from THA and expectations vary according to patients’ severity of condition and their socioeconomic characteristics [3, 4].

Elective procedures such as THA require consent for treatment, which must be informed and voluntary. Discussing with their clinicians is one way for patients to set their expectations, however there are few tools to make this conversation user-friendly and help convey the right information [5]. At best, patients are informed based on clinicians’ experience and by accessing material online, which put them at risk of receiving inaccurate messages, as well as missing information considering their personal characteristics. Arthroplasty registries are a valuable source of information about benefits of THA and the long-term risks of complications, but they are rarely used to support meaningful discussion between patients and their clinicians.

As part of a broader research programme, we developed “Patients like me”, a tool based on registry data that uses baseline and outcome information from previous participants of the Geneva Arthroplasty Registry (GAR) to inform the discussions between prospective patients and their surgeons. The tool was applied to specific outcomes that were considered relevant to patients undergoing THA: pain, activity, complications, and contralateral surgery. Details of the full methodology underpinning the development of the tool are available in a separate publication [6].

In this study we report the methodology and results from “Patients like me” when applied to activity outcomes and show how the tool can support patients expecting to undertake a THA by better understanding how previous patients “like them” did.

## Patients and methods

The primary source of data was the GAR, which collects baseline and long-term follow-up information from participating patients undergoing primary or revision THA at the Division of Orthopaedics and Trauma Surgery of the Geneva University Hospitals (HUG) since 1996. The hospital is the largest in Switzerland and the only public hospital in the State (Canton) of Geneva. Baseline characteristics of the patients in our study are similar to those of the patients in the Swiss National Hip & Knee Joint Registry [7]. Details about the Registry and the information it collects have been published elsewhere [8].

Patients who underwent a primary elective THA between March 1996 and December 2019 were included in the analysis. Participants who had a large head (> 28 mm), metal-on-metal bearing or a bilateral operation on the same day were excluded.

Written informed consent (opt-out) was obtained from the patients before entering the registry for the use of their data for research purposes. Ethics approval for this project was obtained from the Cantonal Ethics Committee for Research in Geneva (Commission Cantonale d’Éthique de la Recherche de Genève (CCER)), under reference n° PB_2017 − 00164.

### Outcomes and predictors

To identify relevant and suitable activity outcomes, a survey was designed through a combination of interviews with patients, insight from hip surgeons, preoperative education sessions and scoping of the published literature. The survey covered several topics including independence in walking, returning to daily activities, resuming their social life, and emotional well-being. 72.6% of a random sample of 379 patients chosen from the GAR registry were sent and completed the survey alongside seven hip surgeons from the Geneva hospital [6]. Based on responses to the survey and insight from both patients and surgeons, five activity outcomes were chosen: the patients’ ability to get in/out of a car, dress themselves autonomously, accomplish their usual tasks, the impact of their physical or emotional problems on their social activities, and their self-reported activity level including activities of daily living, leisure and if applicable professional activity (UCLA Activity score). Predictors of the five outcomes were chosen based on a combination of information from published literature [9], clinical experts, and availability in the registry. Predictors were made up of pre-operative clinical attributes and patient demographics. More details about the outcomes and predictors are given in the Supplementary material (Sect. 1). Each of the five activity outcomes were analysed at one, five-, and 10-years post-surgery in line with the follow-up time points in the GAR. Beyond these activity outcomes, the survey also identified as relevant for both patients and clinicians various pain outcomes, analysed in a recent publication [10], complications such as revision, infection, dislocation, and fracture, and risk of contralateral arthroplasty, which will be reported in a separate publication.

### Statistical analysis

First, overall proportions by response level for each activity outcome at the three follow-up points were reported to show progression for the entire sample from pre-op to 10 years post-surgery.

Classification algorithms were then generated using Conditional Inference Trees (CIT) analysis. CIT was applied to the GAR data as a clustering method (not prognostic) to generate a classification tree for each activity outcome at each of the three time points. The CIT technique employs regression methods to identify statistically significant predictors that progressively break the sample into distinct clusters of outcome levels grouping people highly similar to each other but significantly different to other clusters in terms of their outcomes [11].

CIT tests the global null hypothesis of independence between any of the predictors and the outcome and selects the input variable with strongest association as measured by a p-value. When the association is found to be significant (*p* < 0.05), the corresponding cut-off value splits the node of the current subsample into two sub-branches (child nodes) with significantly different outcome values. This results is a decision tree that grows as more and more predictors are found to be significant and more splits are added, until the test cannot find any predictor that leads to significantly different child nodes [12]. At the end of each final node sits a unique cluster of patients defined by the pathway of significant predictors that led to that node, made up of a unique subgroup of the population sharing similar outcomes but different from those of the patients falling under other terminal nodes.

Not all participants would be expected to provide data for all baseline predictors or outcomes at each time point. Main reasons for missing data include patients choosing not to answer questions, patients moving outside of Switzerland and hence outside of the GAR’s reach for follow-up, patients choosing to stop participating, and potentially patients seeing their general health deteriorate leading them not to complete follow-up questionnaires. These missing data were handled and their impact mitigated by applying multiple imputation methods as detailed in the Supplementary material (Sect. 2). The CIT analysis was performed for each activity outcome and time point except for the SF12 independence outcome at year 10 as there were insufficient observed values to impute missing ones for this variable.

Internal validity was undertaken by generating 1000 bootstrap samples of equal size to the original sample and then repeating the analysis for each. We compared the predictors found to be significant in the primary analysis against those in the 1000 bootstrapped trees using a threshold of 50% to categorise “likely” (“unlikely”) predictors, defined as those found in at least (less than) half of the bootstrapped CITs. Further details are reported in the Supplementary material (Sect. 3).

Lastly, we provide an exemplar of the personalised trajectory that a new patient might observe showing how previous patients like them performed on the five activity outcomes at the three time points by matching their pre-operative predictors to the corresponding clusters.

R software v.4.0.3 and the ‘ctree’ function part of the party-kit R package [12, 13] were used to conduct the analysis.

## Results

The sample analysed was comprised of a total of 6,836 operations. The mean age of patients included in the sample was 68.9 years (SD = 12.2). There were slightly more women (56.8%) than men and almost everyone in the sample had no previous hip surgery (93.1%). Patient characteristics are described in more detail elsewhere [6].

**Fig. 1:**
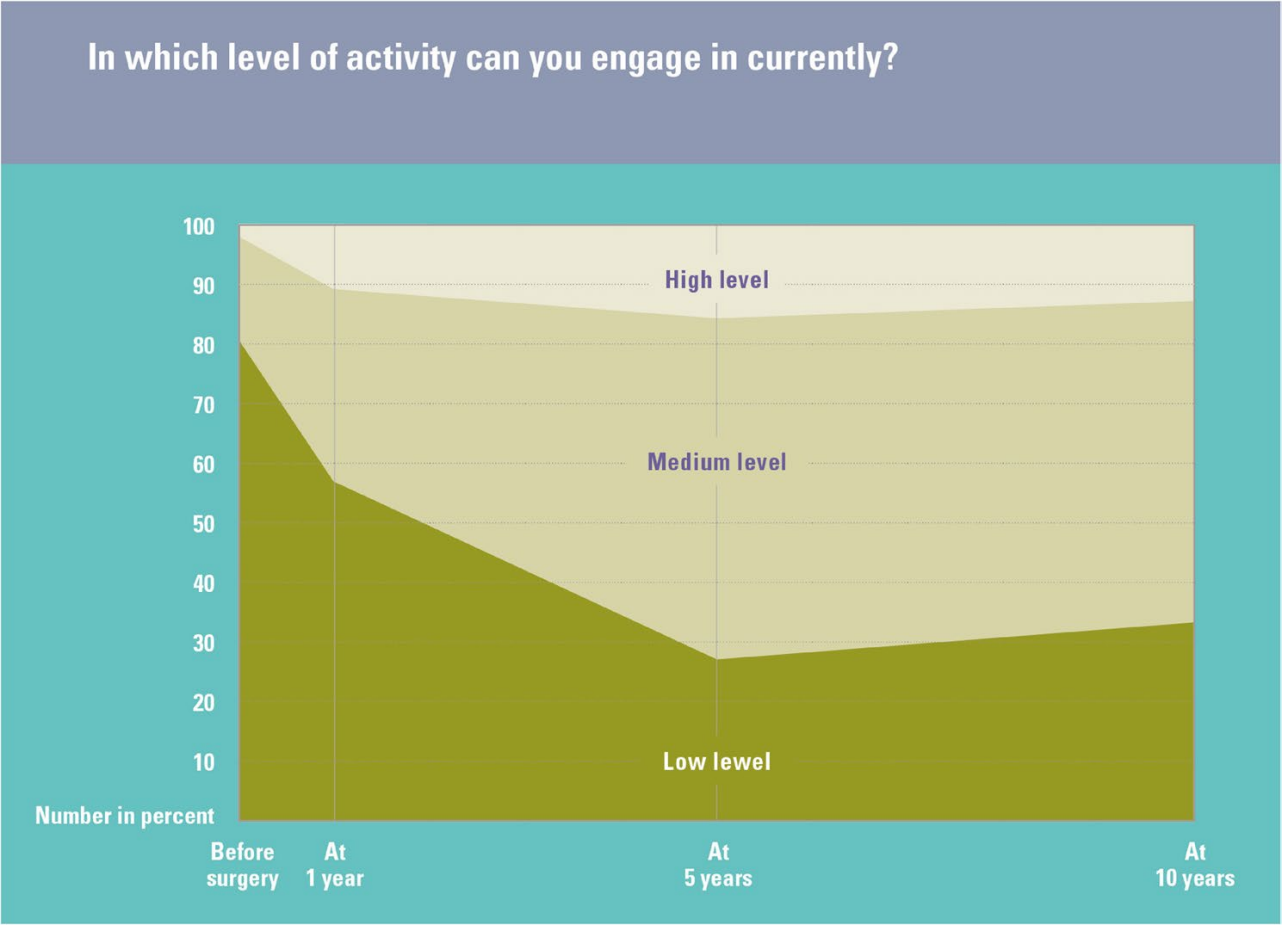
Activity outcome levels (UCLA) before and after surgery (all patients)

Reported activity levels for all activity outcomes measured before surgery and at one, five and 10 years after surgery are shown in Table 1. Across the 10 years of follow-up, all the activity outcomes improved. Figure 1 shows an example of activity outcome levels before and after surgery. The percentage of missing data across the four time points and five activity outcomes ranged from 24.78 to 45.62% (see Table 1).

**Table 1:** Activity level outcomes at baseline and post-operatively

Activity outcomes	number (%)			
	Baseline (pre-op)	1-year post-op	5-years post-op	10-years post-op
Physical independence (SF12– question 4)				
“Have you accomplished less than you would have liked?”				
Yes	3293 (85.27)	755 (38.80)	1279 (44.16)	623 (47.16)
No	569 (14.73)	1191 (61.20)	1617 (55.84)	698 (52.84)
Ineligible	1608	4249	2373	4534
Missing	1366 (26.13)	641 (24.78)	1567 (35.11)	981 (42.62)
Physical interference (SF12– question 12)				
“Over the past 4 weeks, have there been times when your state of health, physical or emotional, has hampered your social life and your relationships with others, your family, your friends, your acquaintances?”				
None of the time	761 (19.90)	717 (36.96)	928 (32.26)	400 (30.51)
A little of the time	684 (17.89)	515 (26.55)	697 (24.23)	326 (24.87)
Sometimes	1366 (35.72)	496 (25.57)	882 (30.66)	421 (32.11)
A good part of the time	815 (21.31)	174 (8.97)	299 (10.39)	123 (9.38)
All of the time	198 (5.18)	38 (1.96)	71 (2.47)	41 (3.13)
Ineligible	1608	4249	2373	4534
Missing	1404 (26.86)	647 (25.01)	1586 (35.54)	991 (43.05)
Activity level (UCLA)				
“Check one box that best describes current activity level.”				
Low	1378 (80.49)	988 (56.75)	548 (26.88)	401 (33.11)
Medium	297 (17.35)	563 (32.34)	1168 (57.28)	654 (54.00)
High	37 (2.16)	190 (10.91)	323 (15.84)	156 (12.88)
Ineligible	4319	4244	3455	4609
Missing	805 (31.98)	851(32.83)	1342 (39.69)	1016 (45.62)
Getting in/out of the car (WOMAC– question 9)				
“How much difficulty do you experience: Getting in and out of an automobile?”				
None	56 (1.48)	822 (42.28)	943 (33.25)	403 (30.93)
Slight	292 (7.71)	571 (29.37)	761 (26.83)	347 (26.63)
Moderate	1210 (31.97)	372 (19.14)	744 (26.23)	344 (26.40)
Severe	2227 (58.84)	179 (9.21)	388 (13.68)	209 (16.04)
Ineligible	1608	4242	2364	4530
Missing	1443 (27.60)	650 (25.06)	1636 (36.58)	1003 (43.50)
Getting dressed autonomously (WOMAC– question 10)				
“How much difficulty do you experience: Putting on stockings or socks?”				
None	153 (4.07)	829 (42.73)	946 (33.31)	409 (31.46)
Slight	267 (7.10)	479 (24.69)	729 (25.67)	315 (24.23)
Moderate	860 (23.68)	361 (18.61)	637 (22.43)	299 (23.00)
Severe	2448 (65.14)	271 (13.97)	528 (18.59)	277 (21.31)
Ineligible	1608	4242	2364	4530
Missing	1470 (28.28)	654 (25.21)	1632 (36.49)	1006 (43.63)

Figure 1 caption: Graphic representation of the proportion of the baseline, 1-, 5-, and 10-year post-op cohorts reporting the three possible levels of activity in response to the UCLA Activity question asking respondents to “check [the] box that best describes current activity level.”

### Conditional inference tree analysis

In total, 14 CITs were generated using 22 variables as significant predictors (out of 27 variables) of activity level at one, five, or 10 years after the elective THA. All activity-related outcomes except for SF12 “Interference” at year 10 resulted in ‘fully grown’ trees, meaning those with more than a single root node.

**Fig. 2:**
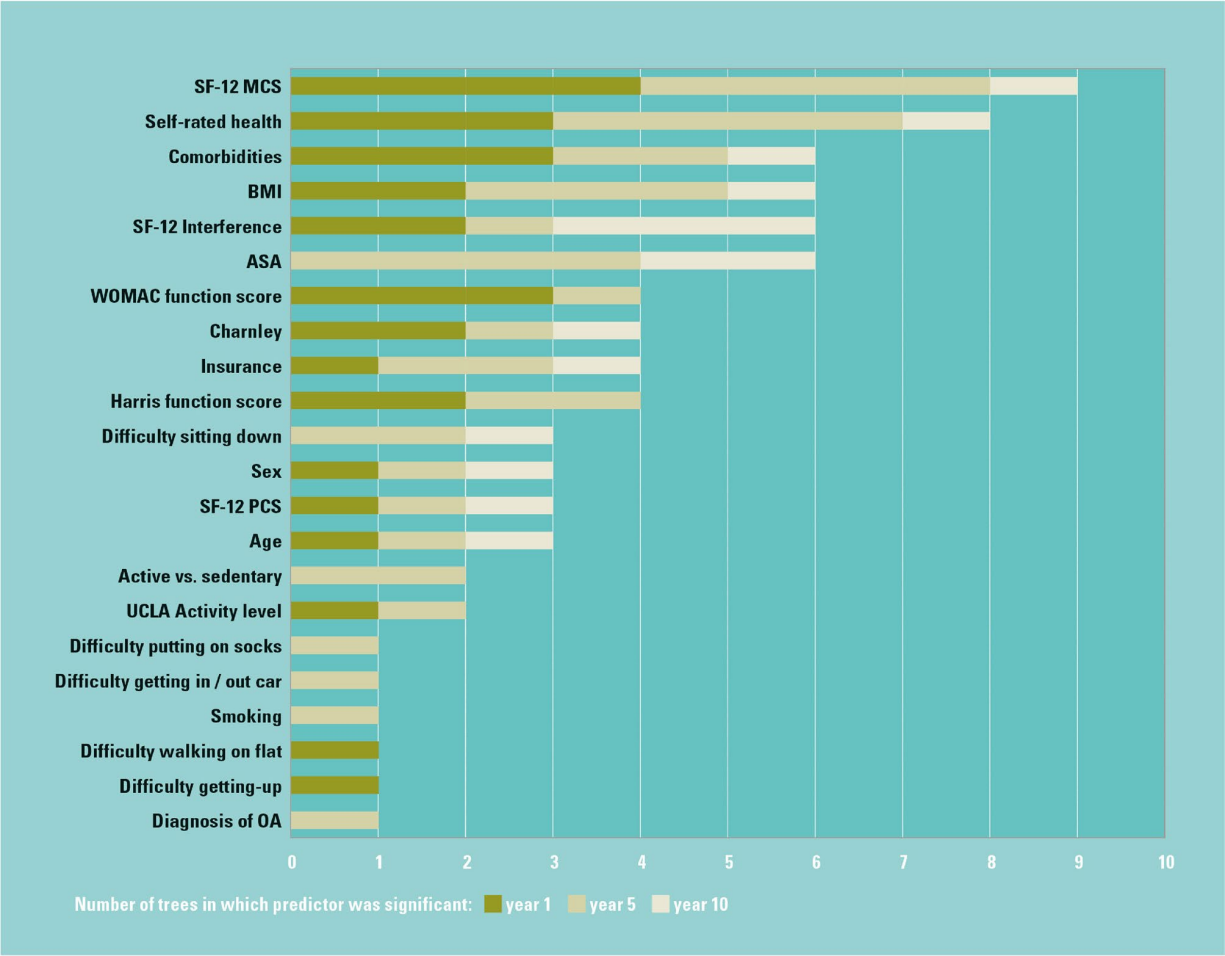
Significant predictors for all activity outcomes by corresponding outcome years

The SF12 MCS, SRH and comorbidity count were the most common predictors determining the trajectory and cluster in which a patient was placed (Fig. 2). At year 1, patients with similar activity outcomes were identified by their overall self-reported mental component score (SF12 MCS), and self-rated health (SRH), as well as the number of comorbidities and the overall WOMAC function score. At year 5, their ASA and BMI also affected the likelihood of a specific activity outcome, whereas at year 10 this was determined by their SF12 interference. Outcome profiles varied markedly by clusters as described in the following sections.

Figure 2 caption: Graphic representation of the number of times each predictor was found significant in all regression trees estimated, by time point, for all five activity outcomes.

#### Independence

Independence during years 1 and 5 was predicted by preoperative SF12 PCS, comorbidity count, Charnley, Harris function, ASA, Sex, BMI, UCLA scale and SRH. Regression trees are shown in the Supplementary material.

At 1 year, 15 nodes (clusters) were identified reporting probabilities of patients accomplishing less in their weekly activities that varied between 9.5% (7/73) and 66.6% (144/216). Patients with higher mental and physical abilities (SF12-MCS and SF12-PCS), fewer comorbidities including orthopaedic comorbidities (Charnley A or B), a lower BMI, greater activity level (Harris function score), and those who are men accomplished as much as they would have liked to in their weekly activities.

At year 5, the proportion of patients having accomplished less than they would have liked ranged between 23.6% (25/106) and 76.4% (71/93) across the eleven clusters. Participants reporting to have accomplished as much as they liked had less orthopaedic comorbidities (Charnley A or B), a higher self-rated health and mental summary (SF12-MCS), ASA equal to 1, and SF12 MCS > 43.1. In contrast, those with ASA ≥ 2, SF12 MCS ≤ 47.3, and Charnley C were more likely to accomplish less than they liked.

#### Interference

The extent by which physical and emotional problems interfered with patients’ social activities was predicted by SF12 MCS, SRH, ASA, type of diagnosis, Charnley, and pre-operative SF12 interference.

At year 1 and 5, seven clusters were identified, whereas at year 10 a single root tree was generated. Probability of patients reporting one year after surgery that physical and emotional problems interfered with their social activities none of the time ranged between 11.3% (15/133) and 82.8% (53/64). At five years this dropped to between 7.1% (7/98) and 80.8% (38/47).

Lower physical interference at year 1 was associated with lower preoperative physical interference, higher self-rated health, a higher mental summary (SF12-MCS), and fewer orthopaedic comorbidities (Charnley A or B). At year 5, in addition to a higher mental summary and SRH, those who reported less physical interference also had lower ASA grade and fewer comorbidities. Figure showing the corresponding regression trees at years 1 and 5 can be found in the Supplementary material.

#### Activity level

At all three time points, the UCLA activity scale was associated with preoperative values of the Harris function and WOMAC scores, WOMAC difficulty getting-up and interference questions, baseline UCLA score, ASA, Activity level, SRH, Insurance, Smoking, Sex, BMI, SF12 PCS, and comorbidity count.

**Fig. 3:**
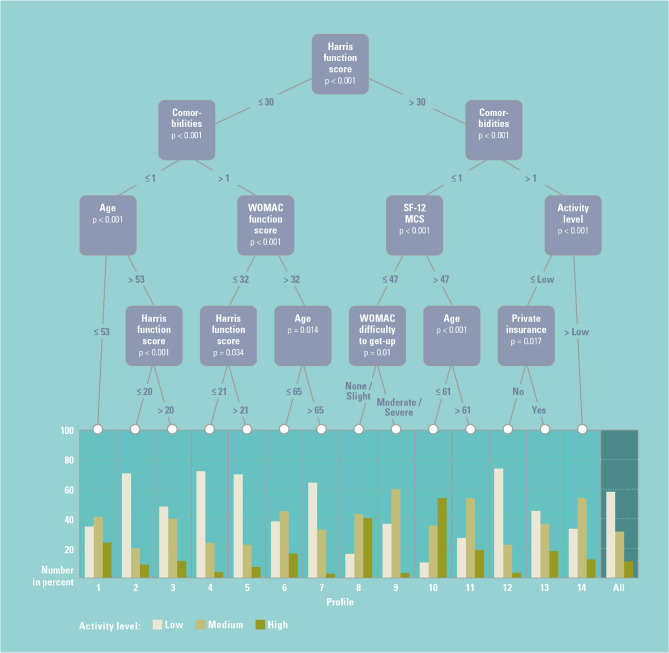
Conditional Inference Tree (CIT) for UCLA activity level at year 1

At year 1 post-op, 14 clusters were identified. The likelihood of patients with “high” UCLA activity level ranged between 2.8% (7/248) and 54.2% (26/48). Patients who reported a higher activity level also had higher Harris function and mental component (SF12-MCS) scores, fewer comorbidities, and younger age. Figure 3 shows the regression tree for UCLA score at year 1 after applying the cut-off points identified in the regression tree to the observed data from registry participants. Trees at years 5 and 10 can be found in the Supplementary material.

Figure 3 caption: Graphic representation of the conditional inference tree for UCLA activity level at year 1. Boxes represent significant (*p* < 0.05) predictors, with the regression identifying thresholds that break the branch into two distinct branches (groups), which are followed by further significant predictors or the final cluster. Clusters are represented by the histogram of the percentage of the cohort at year 1 reporting each of the three levels of activity.

By year 5, CITs identified 23 clusters, with high UCLA score ranging between 1.4% (1/70) and 91.6% (11/12). Participants reporting the greater share of “low” levels of activity in the UCLA scale were older in age, had a lower Harris score, and a greater comorbidity count. Higher levels of activity were reported by patients that were younger in age, with private insurance indicating higher socio-economic status, one or no comorbidities, higher Harris score, and a self-reported preoperative activity level of active or higher.

At 10 years after surgery, 23 clusters were identified. Clusters show between 0% and 52.9% (27/51) of patients reporting high activity scores on the UCLA scale. Patients with the highest UCLA scores were of an age comprised between 49 and 66, with private insurance hence of higher socio-economic status, and fewer orthopaedic comorbidities (Charnley A or B).

#### Getting in/out of the car

The outcome of participants “getting in and out of the car” was most closely associated with preoperative WOMAC function, SF12 MCS, PCS, and interference, SRH, insurance (socio-economic status), ASA, activity level, BMI, and WOMAC “walk” and “sitting down” (questions 8, and 12).

Six clusters were identified at year 1. Proportion of patients reporting no difficulty getting in and out of the car ranged between 20.7% and 53.1%. Highest chances of not having difficulty were observed when patients had a higher WOMAC function score and SRH score of good or higher prior to surgery. Those who had more difficulty getting in and out of the car had lower WOMAC score and SF12 mental component but also severe levels of difficulty while walking (WOMAC). They also reported that their physical health or emotional problems interfered with their social activities at least a little bit of the time.

At year 5, nine clusters were identified. The probability of patients with no difficulty getting in and out of the car by cluster varied between 15.5% and 62.5%. Patients with no difficulty getting in and out of the car had lower ASA, higher SF12 mental component score, and greater SRH scores. The patient cluster reporting difficulty in greater proportion were participants with a greater ASA and BMI.

The tree generated at year 10 had four terminal nodes. Clusters reported no problem getting in/out of a car ranged between 28.5% (107/375) and 56.2% (36/64). Having such problems was most common in patients with baseline higher ASA and moderate to severe difficulty sitting down. The patient cluster reporting difficulty least often had a lower ASA and greater SRH scores. Regression trees at the three time points are shown in the Supplementary material.

#### Putting on socks

The ability to put on socks after years one, five and 10 after THA was associated with predictors such as BMI (an important and consistent predictor throughout all time points), SF12 interference, Harris function, WOMAC questions 9, 10, 12 and the overall score, SRH and comorbidity count.

Five clusters were generated at year 1. Patients rated putting on socks being difficult “none” of the time between 21.7% (17/78) and 64.9% (174/268) across clusters. The predictor trajectory of participants with highest probability of rating putting socks as not difficult was observed in patients with higher SRH and lower BMI. Patients had difficulty putting on socks most often when their SRH score and WOMAC function were lower and had higher comorbidity count.

At year 5 there were nine clusters. The likelihood of clusters with participants finding it not difficult to put on their socks ranged between 22.7% (33/145) and 100% (3/3). Those who had less difficulty putting on socks reported a lower BMI, less physical interference, and a higher Harris score.

By year 10, there were three clusters. The proportion of participants in clusters reporting that putting on socks was not difficult ranged between 24.4% and 35.4%, whilst for a higher degree of difficulty it varied between 15.4% and 33.9% across clusters. Those reporting no difficulty putting on socks had lower baseline BMI, whereas those who rated it as being the hardest had greater BMI alongside SF12 interference being at least “a little of the time”. Corresponding regression trees at 1, 5 and 10 years can be found in the Supplementary material.

### Internal validation

For ten out of the 14 CITs all predictors identified in the main analysis appeared in > 50% of the 100 generated bootstrapped CITs. Of all the predictors appearing in each of the remaining four CITs, no more than two appeared in < 50% of the bootstrapped CITs for each tree. Further details on the validation results are available in the Supplementary material (Sect. 3).

### Personalised trajectories of the five activity outcomes

**Fig. 4:**
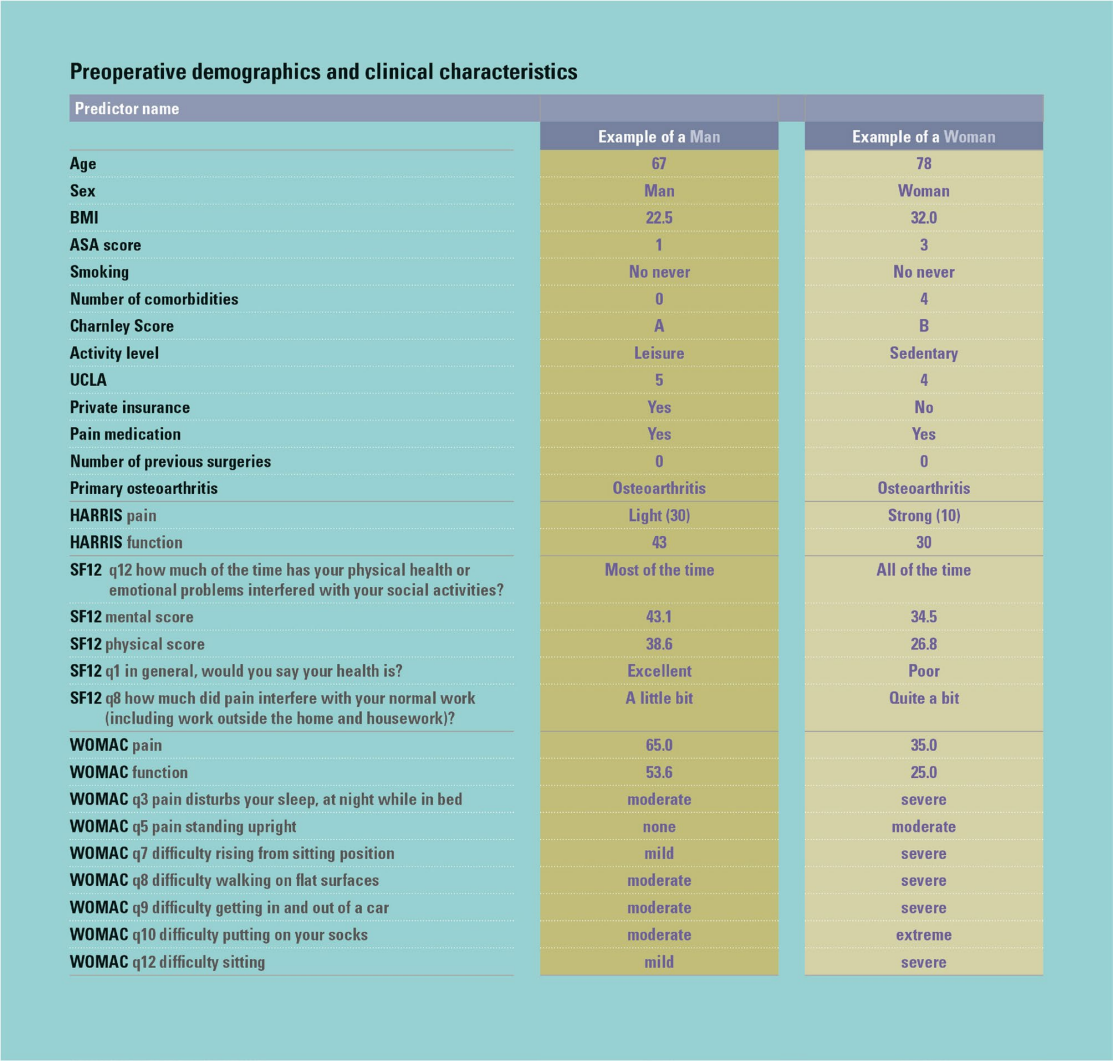
Baseline characteristics for exemplar man and woman patient

**Fig. 5:**
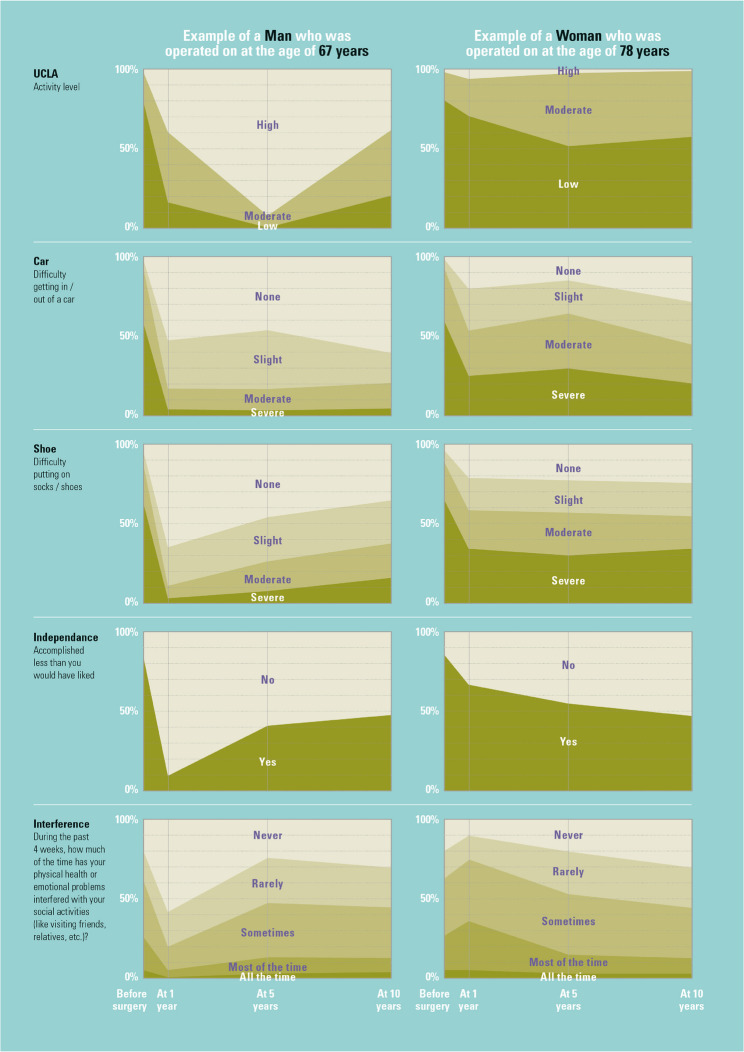
Activity trajectories for ‘patients like’ exemplar man and woman patient

Any patient who answers the corresponding questions for all relevant pre-operative predictors can be matched to a single cluster for each of the five activity outcomes at 1, 5 and 10 years (except interference which generated trees only at 1 and 5) thanks to the creation of the CITs. Figure 4 depicts the baseline characteristics of a hypothetical male and female patient, while Fig. 5 displays their matching clusters.

Figure 4 caption: Characteristics of a hypothetical man and woman just prior to their hip arthroplasty and including all the items necessary to associate the individual to people ‘like them’ who have already had the surgery. With this information, they can be associated to a cluster for each of the five activity outcomes and the reported values of previous ‘patients like them’ shared to inform their pathway.

Figure 5 caption: Values reported by previous ‘patients like’ the exemplar man and woman described in Table 4 for each of the five activity outcomes at baseline, 1-, 5-, and 10-years post-op. Clusters used to generate the values at each point were those corresponding to the hypothetical man and woman according to their baseline characteristics as reported in Table 4.

## Discussion

This analysis was part of an broader study which developed an informational tool on the outcomes of patients following hip surgery based on the experience of previous patients in the GAR [6]. The activity levels of most patients undergoing an elective THA improved substantially after surgery. However, distinct clusters of varying outcome levels can be identified based on information collected before the operation. Results of conditional tree analysis highlighted specific variables and thresholds (cut-off points) that help define such clusters and showed that their relative importance changed at different time points for the same outcome. For example, for WOMAC socks, whilst clusters at year 5 were generated based on BMI, SF12 interference, WOMAC score, WOMAC questions 9, 10, 12, and the Harris score, by year 10 clusters were generated based on baseline BMI and SF12 interference alone. This suggests that whilst baseline BMI, level of interference, the difficulty experienced getting in/out of a car, putting on socks, sitting down, and overall activity capacity are associated with how well activity progresses over the first five years after THA, it will be mainly preoperative BMI and level of interference what will likely predict activity by year 10. This is information not readily available for patients who are soon to undergo a THA, and which is likely to be highly relevant for them as they go through their surgery and post-op recovery.

Most evidence of physical outcomes following a THA are given by mean scores with limited reporting of proportions. From the literature available, unfavourable outcomes following a THA are reported with a range from 7 to 20% [14–16]. Our study falls in line with these results: one year after THA, 14% of registry participants reported having severe difficulty getting dressed autonomously and 11% reported that physical interference impacted them a good part of the time or more. Activity results were best at 1 year and somewhat decreased at 5 and again at 10 years except for the UCLA activity scale, which improved up to 5 years after surgery. Literature on long-term activity trajectories after THA is sparse. Improvement has been reported in the Harris hip score up to 1 year (2 years for the Oxford score) followed by slight deterioration up to 7 [5] years after surgery [17, 18]. Preoperative mental health status, self-rated health, comorbidity count/ASA score, and BMI most often distinguished activity outcome profiles at 1, 5 and 10 years after surgery. Whereas worse mental health, more comorbidities and higher BMI have previously been associated with less activity improvement over the mid-term [17, 18], lower self-rated health is identified for the first time as important predictor of long-term activity outcome. To our knowledge this is the first study that used registry data to develop a tool to inform patients who are about to undergo elective THA and their surgeons about the activity outcomes reported by previous patients like them based on common preoperative characteristics. The novelty of this work is that it uses clustering methods (via regression trees in our analysis) instead of clinical prediction or prognostic models to generate information about what surgery resulted in for patients like them instead of attempting to predict specific outcomes for individual patients.

Previous studies have used large datasets to produce individualised predictions and inform patients about the likely quality of life benefit of surgery [19]. Also, prediction models have gained substantial attention by scholars and they have often been employed to inform clinical practice [20] but often failed to predict the outcomes of orthopaedic surgery [21, 22]. Furthermore, prediction models would result in estimates of the likelihood that a specific patient experiences a particular outcome, commonly explained as a given number in 100 people having such fate [23] which are not straight forward to understand for most patients receiving a THA, much less when they won’t go through 100 iterations of the intervention to make sense of risks or percentages. Although in our study we use regression models, these are employed as a vehicle to identify variables to create clusters, which can be matched to the patient who is about to have surgery based on common preoperative characteristics. By doing this, we can present prospective patients with information about 100 people like them, of whom a known number would have experienced the outcome of interest. We strongly believe this would be more easily understood by patients and help them have meaningful discussions with their clinicians.

It should be highlighted that patients may value different activity capabilities differently from each other. It is therefore important for patients and those who aid in the decision-making process, to consider what activities are most important to them as that may impact their final decision. This information tool could be able to assist and support these considerations.

Our work has some limitations. First, the CIT method identifies cut-off points in the predictors based on the application of a statistical test which, other than changing the p-value, cannot be manipulated. This means that changes in the predictors or the distribution of their values can cause cut-off points and hence final clusters to change in ways that are not only beyond the control of the analyst but that can also be significant given the cascade structure of the regression trees. These may in turn limit the replicability and generalisability of the analysis [24]; however, alternative methods such as Classification and Regression Tree Analysis, although allowing for various criteria to be applied when selecting cut-off points, are more prone to bias by allowing those choices. To assess the impact of this limitation, we conducted an internal validity which confirmed the stability of the split of predictor variables, with 10 of 14 trees retaining all predictors in > 50% of bootstraps (see Supplementary material, Fig. 1). A second limitation is the loss of precision in our findings due to the high levels of missing data, which increase with follow-up time and participants’ age [7], resulting in missing data rates as high as 45% at the 10-year mark. High levels of missing data and hence fewer complete observations for longer-term outcomes may lead to unstable clusters if CITs have numerous splits and terminal nodes end with fewer complete observations. Observing high rates of missing data in studies with long-term real-world follow-up is common, and in fact consistent with those of a previous study reporting results at 7-years of follow-up [17]. Missing data was investigated by using logistic regression to check for bias and imputed where appropriate (see Supplementary material, Tables S2-S25). Additionally, to minimise bias from missing data, the distribution of each outcome variable was determined using observed data. Undertaking multiple imputation by chained equations using the large breath of baseline predictors available in the GAR mitigated the impact of reduced precision and unstable clusters due to missing data. Finally, our study used data collected by a registry of patients from a single hospital in Geneva, which limits the generalisability of our findings. More research is therefore needed to confirm and externally validate our findings in populations in other settings, which should include criterion validity, construct validity, and reliability.

It should be highlighted that this tool is for informational purposes only and is not a prognostic tool. A prognostic model is a mathematical equation which calculates the probability of a particular outcome using multiple predictors from the individuals known information [25]. This means a prognostic model can be validated by comparing the observed and predicted outcomes. As our information tool clusters individuals who have already undergone a total hip arthroplasty for which new patients can be matched against to give a reference point, it cannot be validated in the same way. It is therefore recommended that caution is advised for clinicians using this tool, emphasising that it is an information tool and not prognostic. The information tool includes a patient information leaflet, a digital visualization tool for surgeons, and an infographic brochure to improve user-friendliness for both patients and clinicians although formal evaluation of users’ experience has not yet been conducted.

This study has a wide range of implications. Firstly, the information tool will help and guide discussions between patients and surgeons at HUG to ensure that, prior to the surgery, the patient has clear expectations of their outcomes following the operation. An external validation would assess its generalisability beyond the HUG and the patient population captured by the registry; however, these validations are likely to include only a subset of the outcomes and predictors used in this analysis given the unusually large number of variables collected by the GAR, their granularity and length of follow-up. In the meantime, the findings should be interpreted within the context of clinical consultations that may lead to surgery at the HUG.

Secondly, this information is relevant to hospital providers to better understand the likely physical outcomes of the patients, enabling them to adjust their future care plans accordingly. Thirdly, this study provides a methodology which can be applied to other registries and elective operations such as a total knee arthroplasty.

## Conclusions

Data registries like the GAR can provide a wealth of information which, using the information tool we have developed, can inform patients about the outcomes of people like them and the timeline of potential progress following surgery. As this tool was developed using data from a single institution and long-term outcomes rely heavily on imputed data, it should be interpreted and used with caution until external validation, even if partial, is undertaken to inform broader implementation. This information tool can be used to aid both meaningful discussions between the patients and clinicians regarding physical outcome and help design their care plans going forwards.

## Electronic supplementary material

Below is the link to the electronic supplementary material.

Supplementary Material 1

## Data Availability

The datasets generated and/or analysed during the current study are not publicly available due to local data protection rules, but de-identified data are available from the corresponding author on reasonable request and after permission from the local ethics committee.
