# Supplementary material for: Activity outcomes after hip arthroplasty: an information tool based on patients’ experience captured in a hospital registry
Source: BMC Musculoskelet Disord. 2025 Aug 20;26:804. doi: 10.1186/s12891-025-09024-w (PMC12366395; doi:10.1186/s12891-025-09024-w)
Supplement: Supplementary file 1 — Supplementary Material 1 [file 12891_2025_9024_MOESM1_ESM.docx]

## **Outcomes and predictors:**

The specific outcome list matched the Outcome Measures in Rheumatology Trials (OMERACT) core domain and included the Western Ontario and McMaster Universities Arthritis Index (WOMAC) questions measuring ability to get in and out of the car and putting on socks. Due to the limited differentiation between responses of “extremely severe” and “severe” in the WOMAC questionnaire we merged these responses into one group called “severe”. The SF12 questions about how much their physical health or emotional problems interfered in their social activities and whether they accomplished less in weekly activities (independence), as well as the UCLA activity score were also included in the analysis.

A set of demographic and activity specific variables was used as predictors. We included age, sex, BMI, comorbidity count and previous hip surgeries, type of diagnosis, symptom duration, ASA grade, Charnley disability grade (abbreviated as Charnley in the following), smoking. As a proxy for socioeconomic status we used a variable reporting whether participants had public or private health insurance [5]. Moreover, we used activity specific variables measured at baseline such as: SF12 questionnaire (questions 1, 4, 12, physical component score (PCS) and mental component score (MCS)), WOMAC questionnaire (questions 6,7,8,9,10,11,12 and the function score), Harris function score, UCLA activity score and a measure of activity levels (see Supplementary material for a detailed description of each predictor). Table S1: Predictors and baseline values

| **Outcomes** | **Description** |
| --- | --- |
| Physical independence | SF12 – question 4: “Have you accomplished less than you would have liked?” |
| Physical interference | SF12 – question 12: “Over the past 4 weeks, have there been times when your state of health, physical or emotional, has hampered your social life and your relationships with others, your family, your friends, your acquaintances?” |
| Activity level | UCLA: “Check one box that best describes current activity level.” |
| Getting in/out of the car | WOMAC – question 9: “How much difficulty do you experience: Getting in and out of an automobile?” |
| Getting dressed autonomously | WOMAC – question 10: “How much difficulty do you experience: Putting on stockings or socks?” |
| **Predictors (baseline values)** | **Description** |

| Age | Age of participants on the day of the surgery |
| --- | --- |
| Sex | Female/Male |
| BMI | Body mass index - Quetelet formula (kg/m2) |
| ASA grade | ASA Physical Status Classification System (healthy, mild systemic disease, server systemic disease, severe systemic and threat to life) |
| No. of comorbidities | Number of recorded comorbidities |
| Charnley | Charnley classification (A: patients have single joint arthropathy and no significant medical comorbidity, B: patients have one other joint in need of an arthroplasty, or an unsuccessful or failing arthroplasty in another joint, C: patients have multiple joints in need of arthroplasty, multiple failing arthroplasties or significant medical or psychological impairment) |
| Diagnosis | Number of diagnoses (underlying condition) |
| Smoking | Whether patients smoked (never, former, current) |
| Insurance | Whether patients had private insurance at time of surgery |
| No. of previous surgeries | Number of previous surgeries recorded in GHAR |
| OA symptom duration | Duration of symptoms in years (<1 year, 1-2 years, 2-5 years, >5 years) |
| Self-rated health | SF-12 – question 1: “Overall, do you think your health is?” (Poor, fair, good, very good, excellent) |
| Physical independence | SF12 – question 4: “Have you accomplished less than you would have liked?” |
| Physical interference | SF12 – question 12: “Over the past 4 weeks, have there been times when your state of health, physical or emotional, has hampered your social life and your relationships with others, your family, your friends, your acquaintances?” |
| Physical Composite Scale | SF-12 - Physical Composite Scale – PCS |
| Mental Health Composite Scale | SF-12 - Mental Health Composite Scale - MCS |
| Physical Function - stairs | WOMAC – question 6: “How much difficulty do you have: climbing stairs” |
| Physical Function – getting up | WOMAC – question 7: “How much difficulty do you have: getting up from a sitting position” |
| Physical Function – walking | WOMAC – question 8: “How much difficulty do you have: walking on level ground” |
| Physical Function – car | WOMAC – question 9: “How much difficulty do you have: entering and exiting a car” |
| Physical Function – socks | WOMAC – question 10: “How much difficulty do you have: putting on stockings or socks” |
| Physical Function – bed | WOMAC – question 11: “How much difficulty do you have: getting out of bed” |
| Physical Function – sitting up | WOMAC – question 12: “How much difficulty do you have: sitting up” |
| WOMAC function score | WOMAC overall score, 0-100 (=no function) |
| Harris hip function | Harris hip function sub score, 0-47 (=no function) |
| Activity level | “Sedentary”, “Active”, “Sports/leisure" or "Competition" |
| Activity level (UCLA) | "Low", "Medium", "High" |

## **Missing data**

Multiple imputation was used to mitigate the impact of missing data on results due to loss to follow-up over time. Missing data was imputed on BMI, comorbidity count, Charnley score, smoking status, number of previous hip surgeries, OA symptoms duration, and questionnaire items (SF12, Harris Function Score, WOMAC). ASA grade, diagnosis, and private insurance showed no missing data hence there was no need to impute.

First, to ascertain whether there was any association between the observed variables and the missing data logistic regressions were ran on the outcomes and predictors. As a result, data was considered to be largely missing at random due to variables for which there was complete information (such as age, sex and ASA score) accounting for missingness. Secondly, chained equations methods [12] were applied and the variables with missing data were used as response. Variables that were identified in the logistic regressions were considered as predictors as well as other variables considered potentially associated. For example, an activity outcome would be considered a reasonable predictor for another activity outcome. Lastly, to check there were not distinct differences we compared the observed and imputed data stratified by sex, age, and BMI (see supplementary material, Tables S2-25).

Due to CIT method not being able to manage multiple imputed datasets, to generate the trees we used the first of the 50 imputed data sets and further patients’ allocation in each of the nodes was calculated based on the observational data allowing clusters to be defined with mitigated bias from the missing data.

## **Internal validation**

### Methods:

We compared the predictors which appeared in our original analysis to number of the times each predictor appeared in the 1000 bootstrapped trees for each activity outcome (Figure S1). In Figure S2, we compare the mean size of the trees generated using the bootstrap samples to the size of the tree in the model. The bars in the plot indicate the min-max 95% percentiles.

### Results:

All predictors in 10 of the 14 CITs of the main analysis also appeared in >50% of the 1000 bootstrapped trees generated for validation. In the remaining four CITs, at the most only two (WOMAC car and socks at years 1 and 5) of all predictors identified in the main analysis were found in <50% of the bootstrapped trees.

In the main analysis the tree for SF12 interference at year 10 resulted in a single node; in the validation, this outcome reported only ‘unlikely’ predictors, with the most common appearing in <40% of the bootstrapped trees.

Seven of the CITs in the main analysis had more clusters (terminal nodes) than the average size of the 1000 bootstrapped trees.

A summary of predictors and a comparison of the size of trees after validation is provided in the Supplementary material (Figure S2).

Figure S1: Model Validation, variable frequency


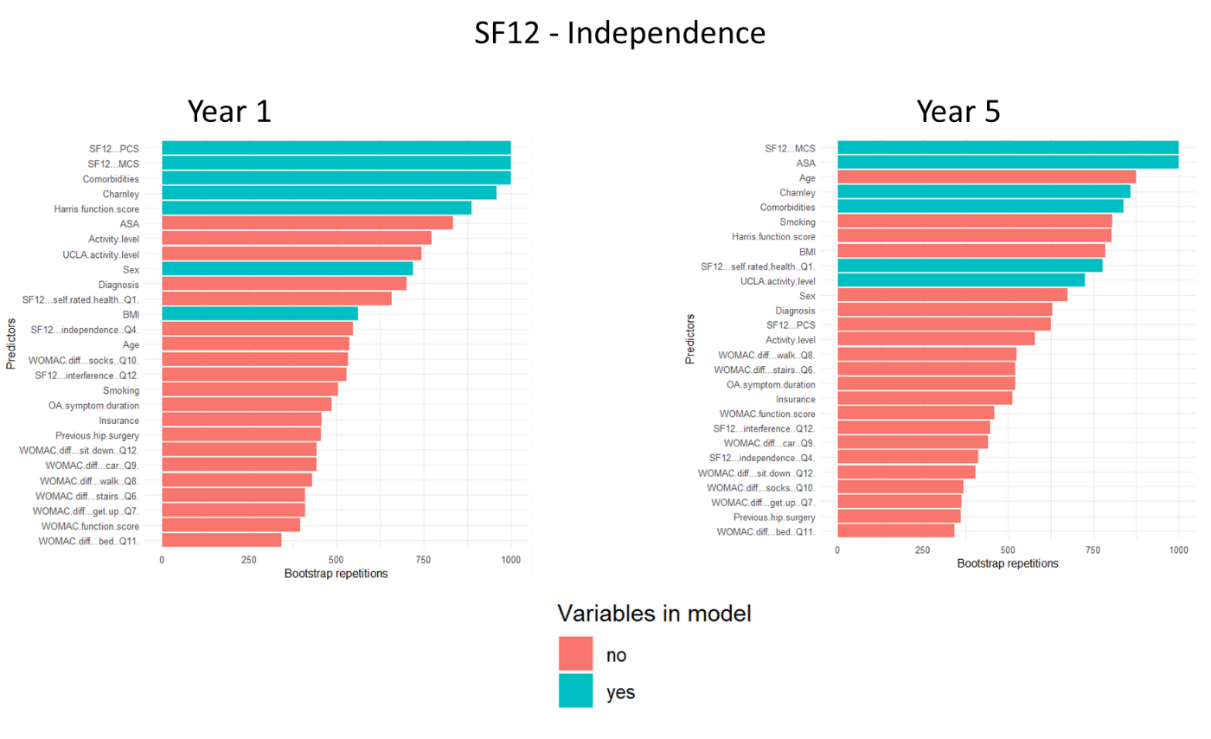


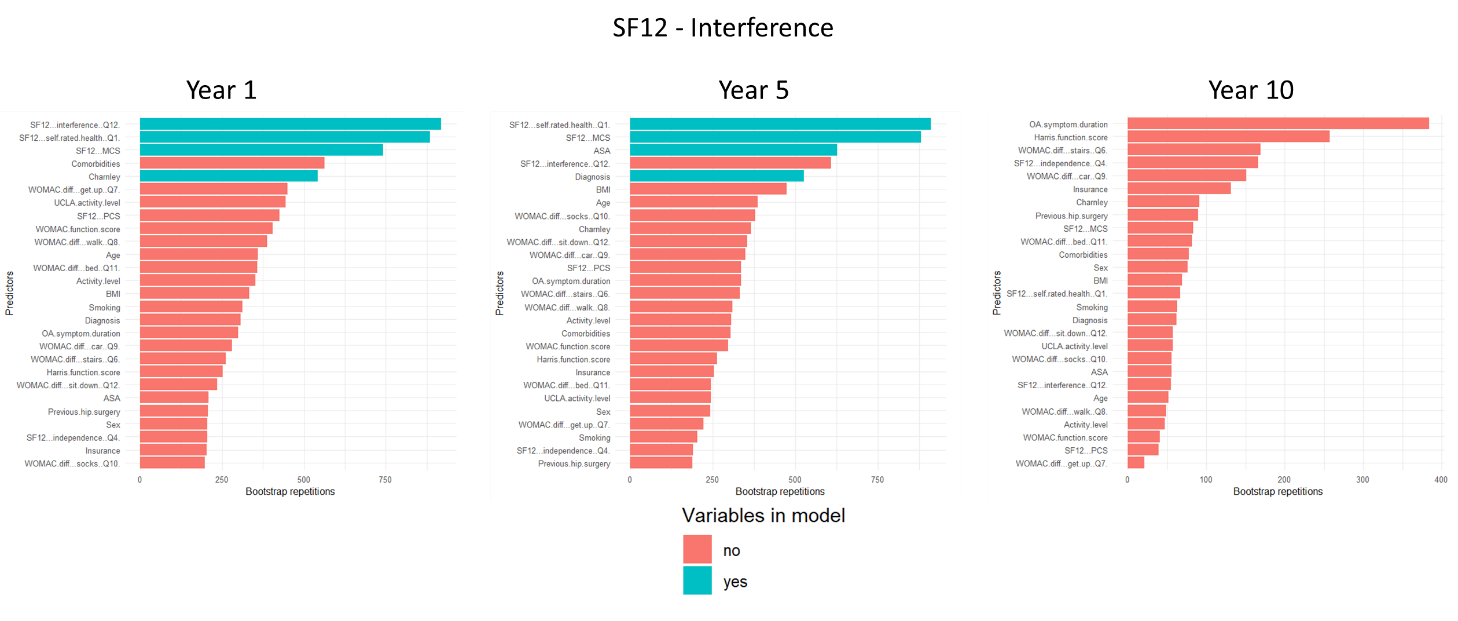


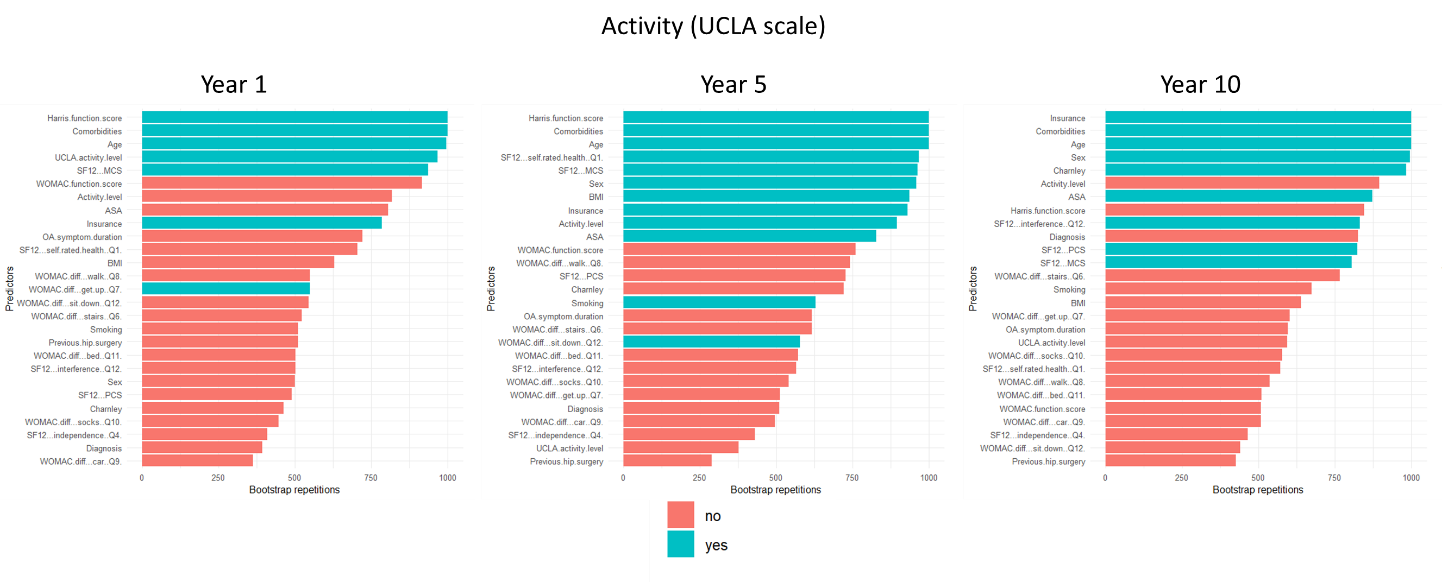


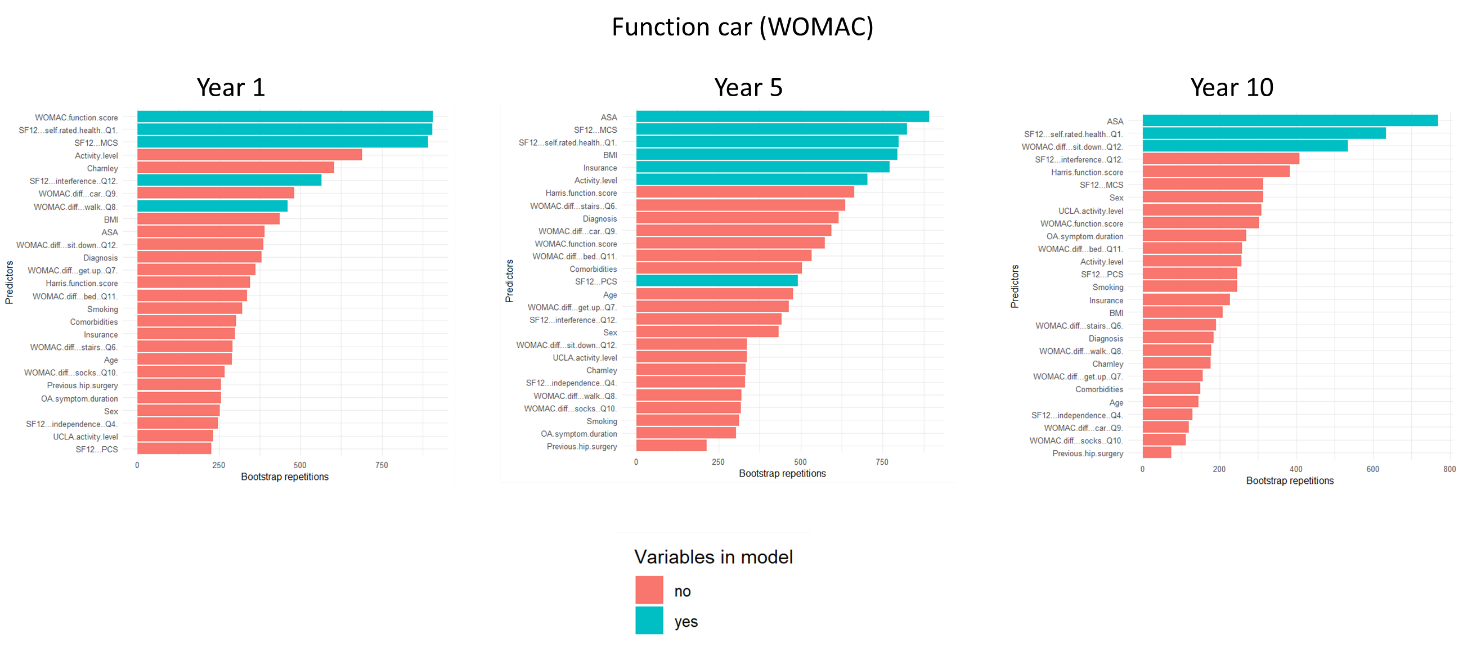


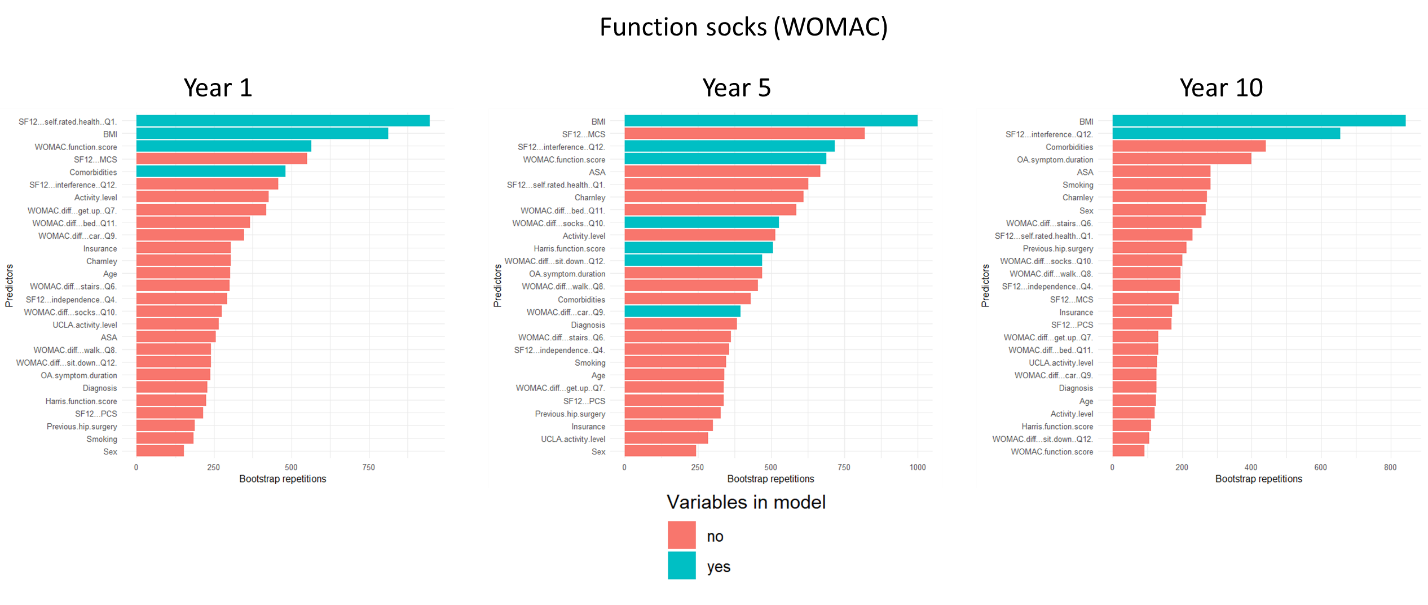


Figure S2: Model validation, tree size


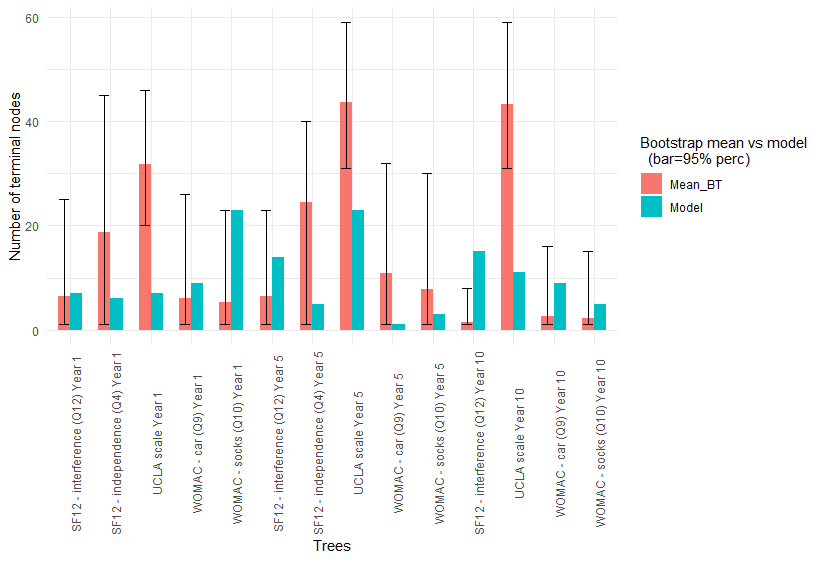


## **Results of Conditional Tree analysis**

Figure S3: Independence - year 1


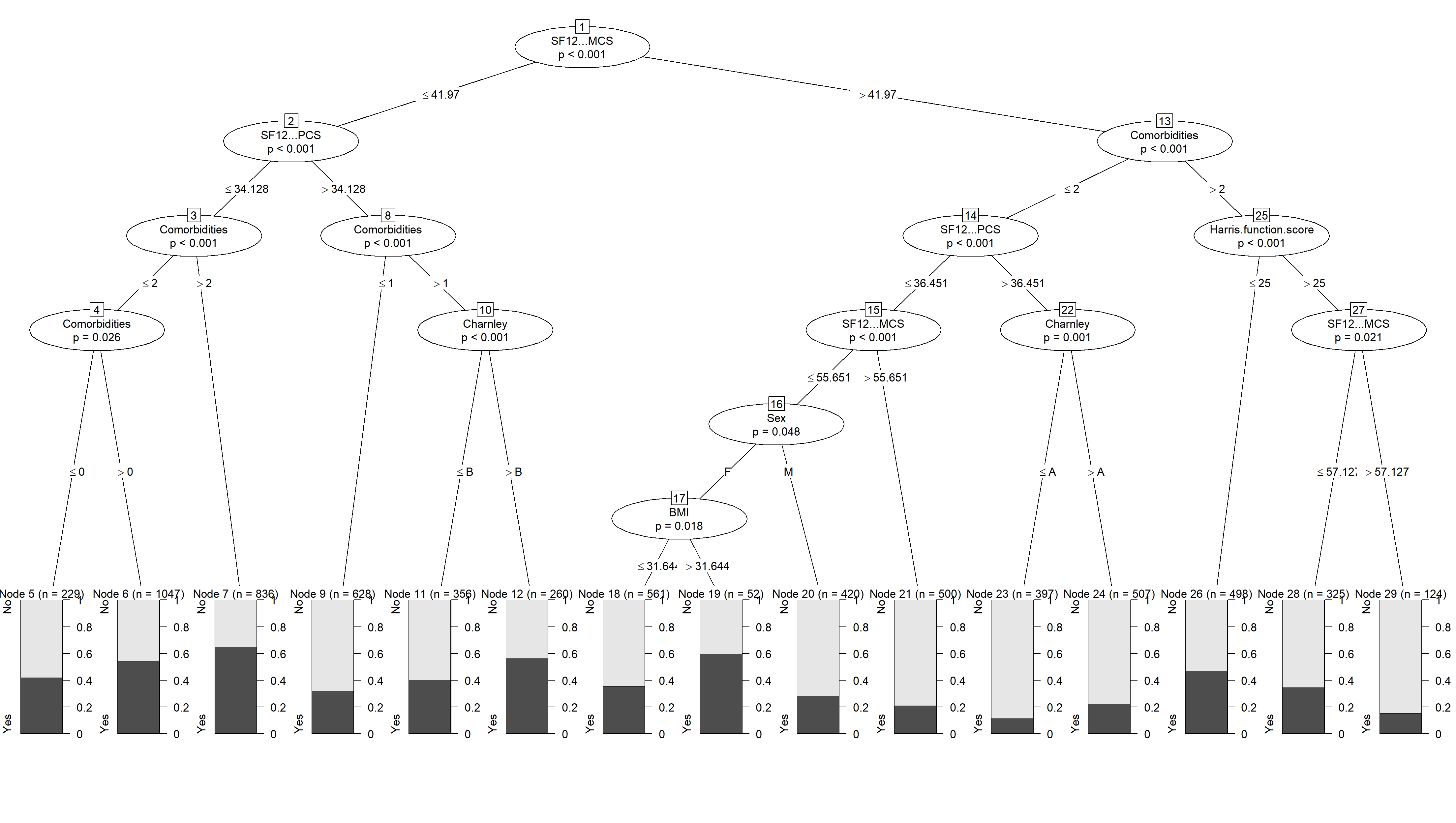


Figure S4: Independence – year 5


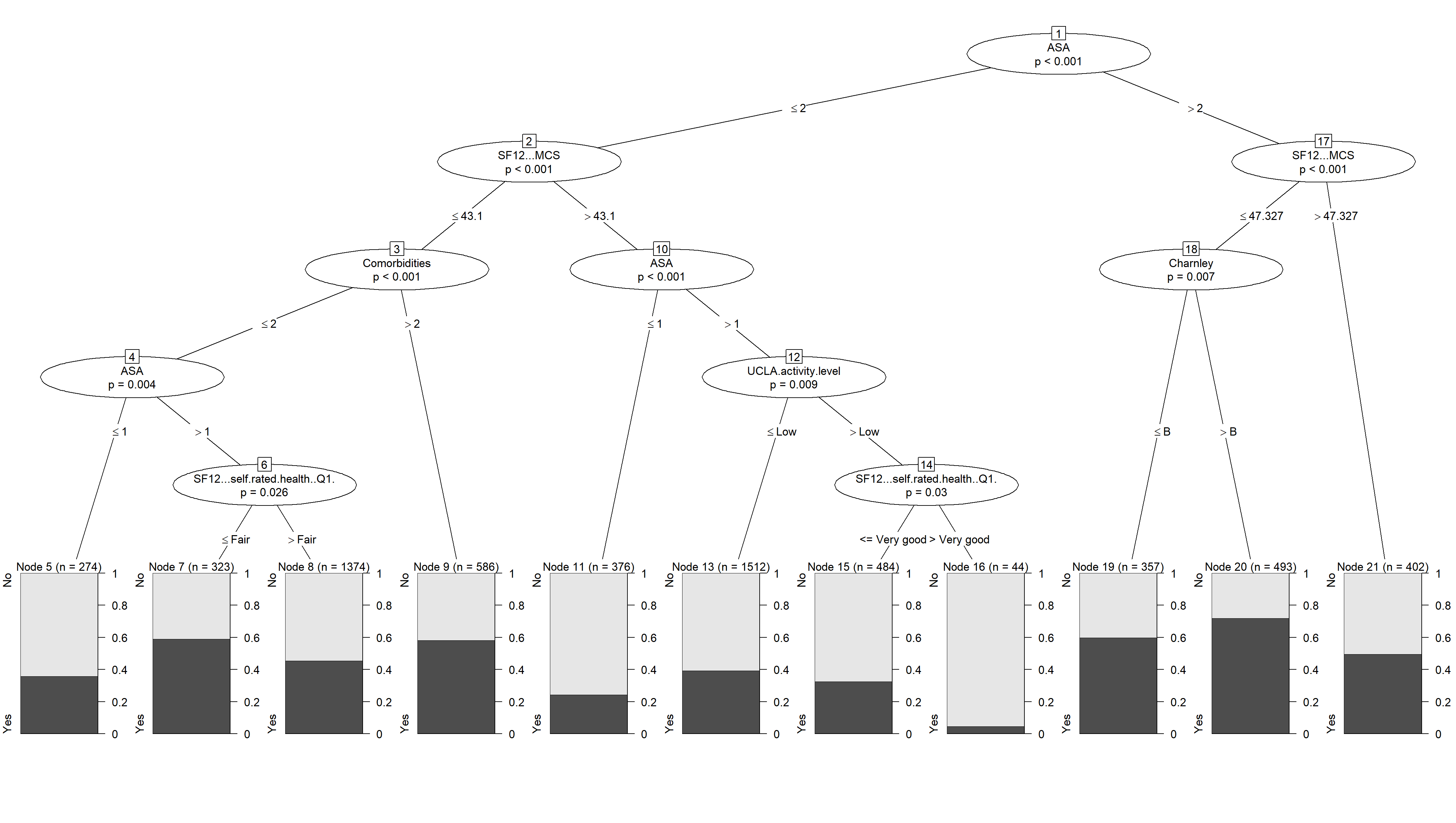


Figure S5: Interference – year 1


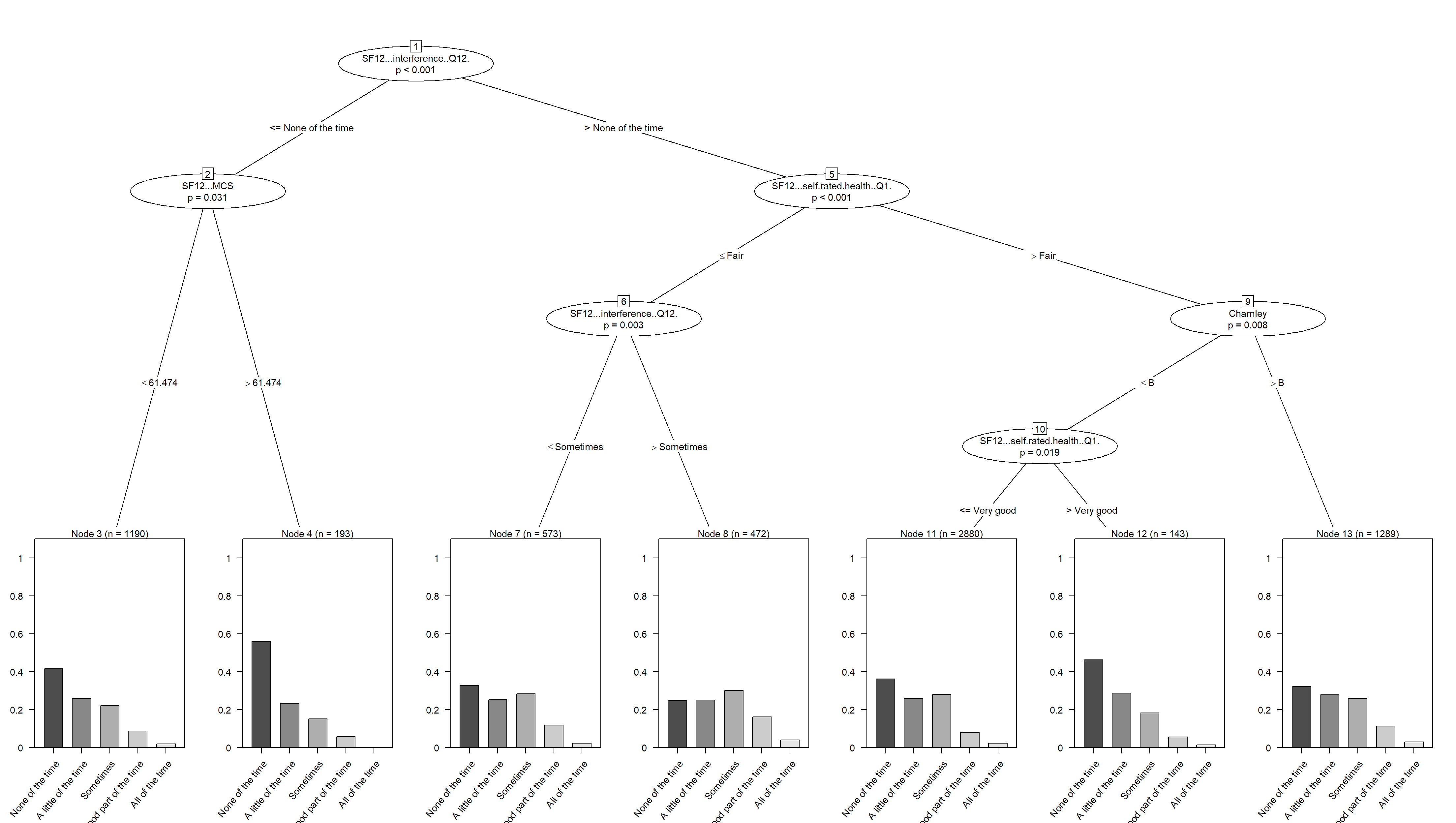


Figure S6: Interference – year 5


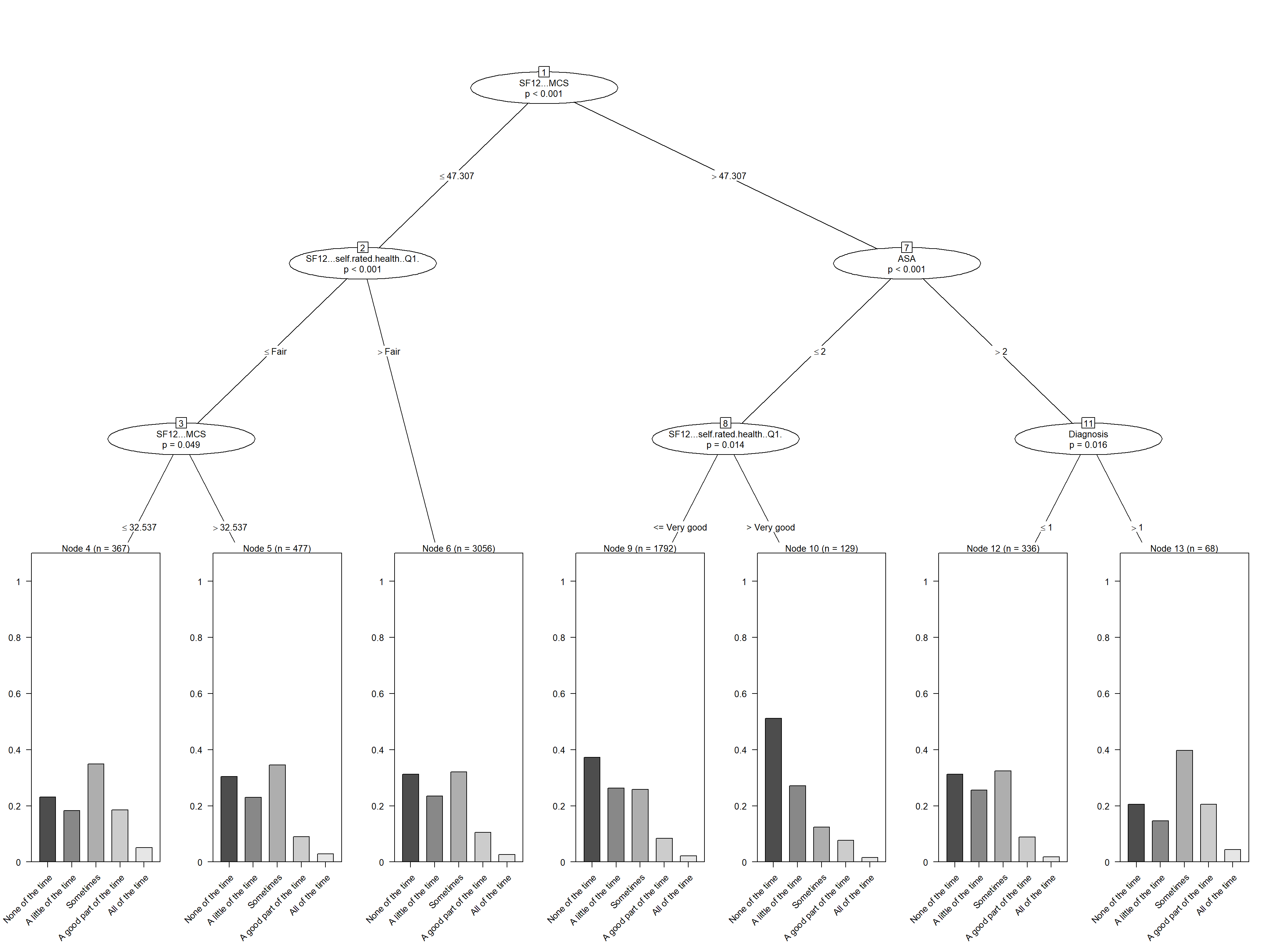


Figure S7 Interference – year 10


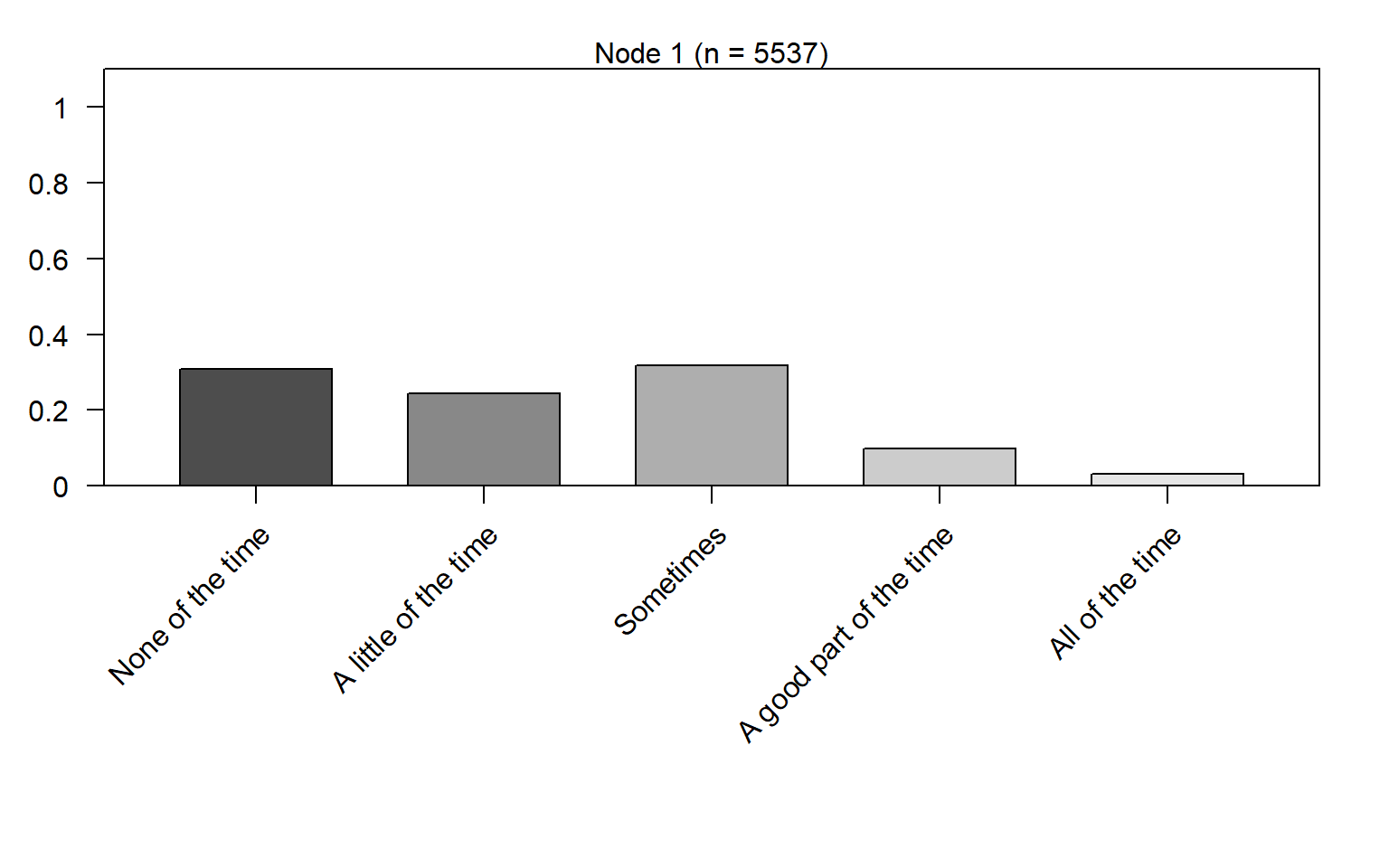


Figure S8: UCLA, activity level – year 1


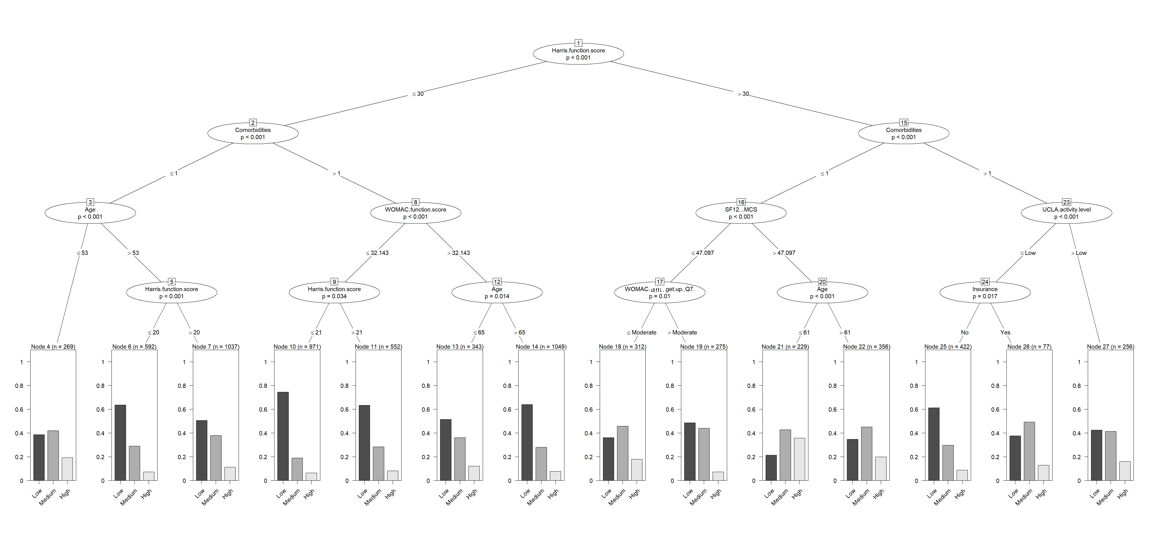


Figure S9: UCLA, activity level – year 5


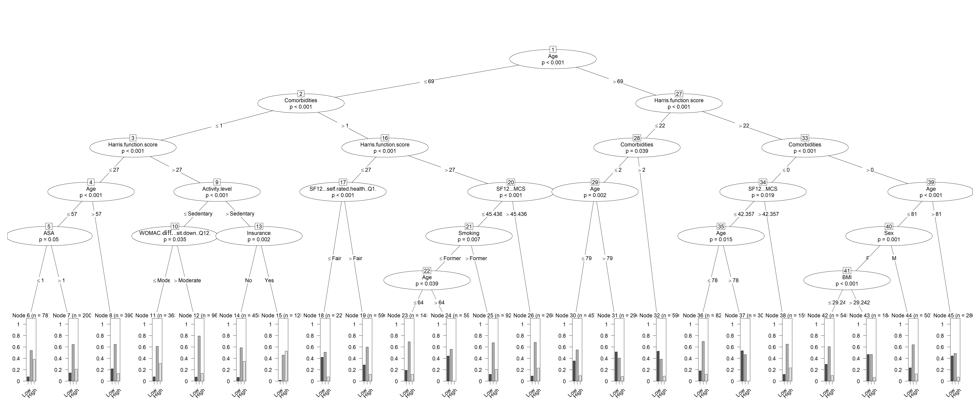


Figure S10: UCLA, activity level – year 10


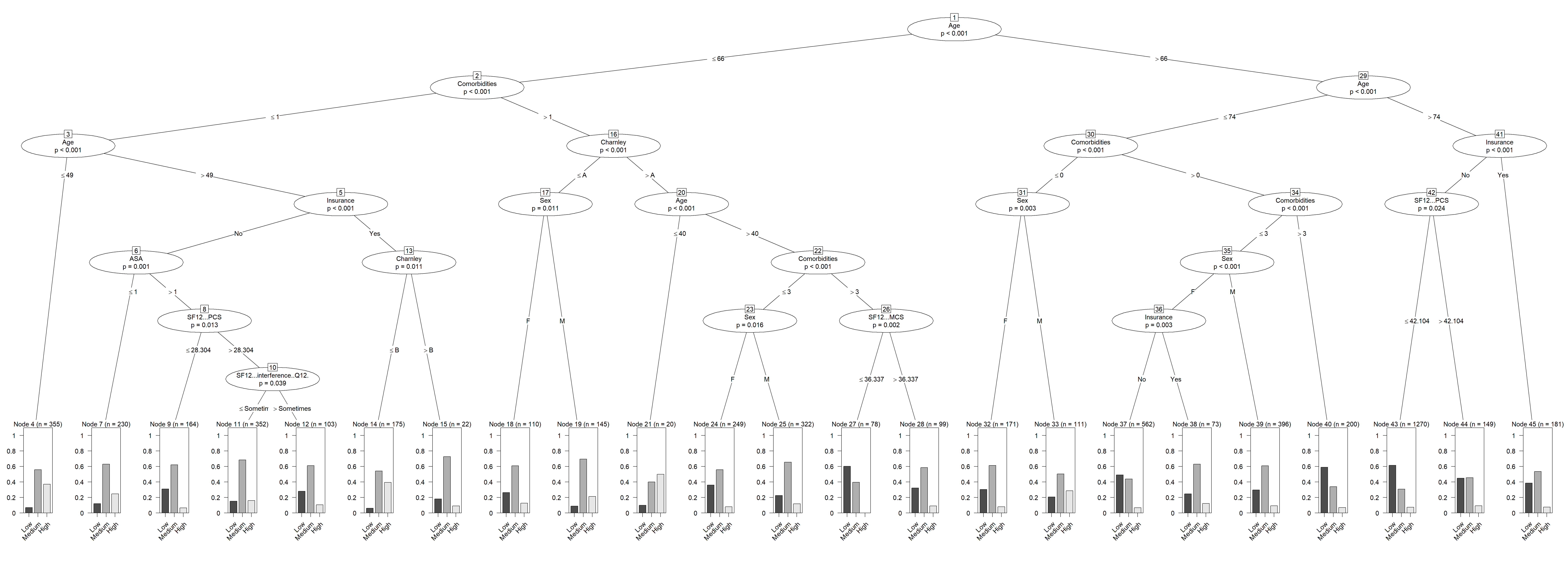


Figure S11: Ability to get in/out of a car – year 1


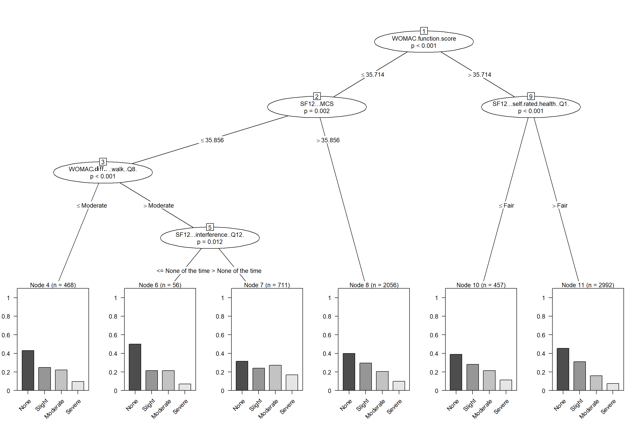


Figure S12: Ability to get in/out of a car – year 5


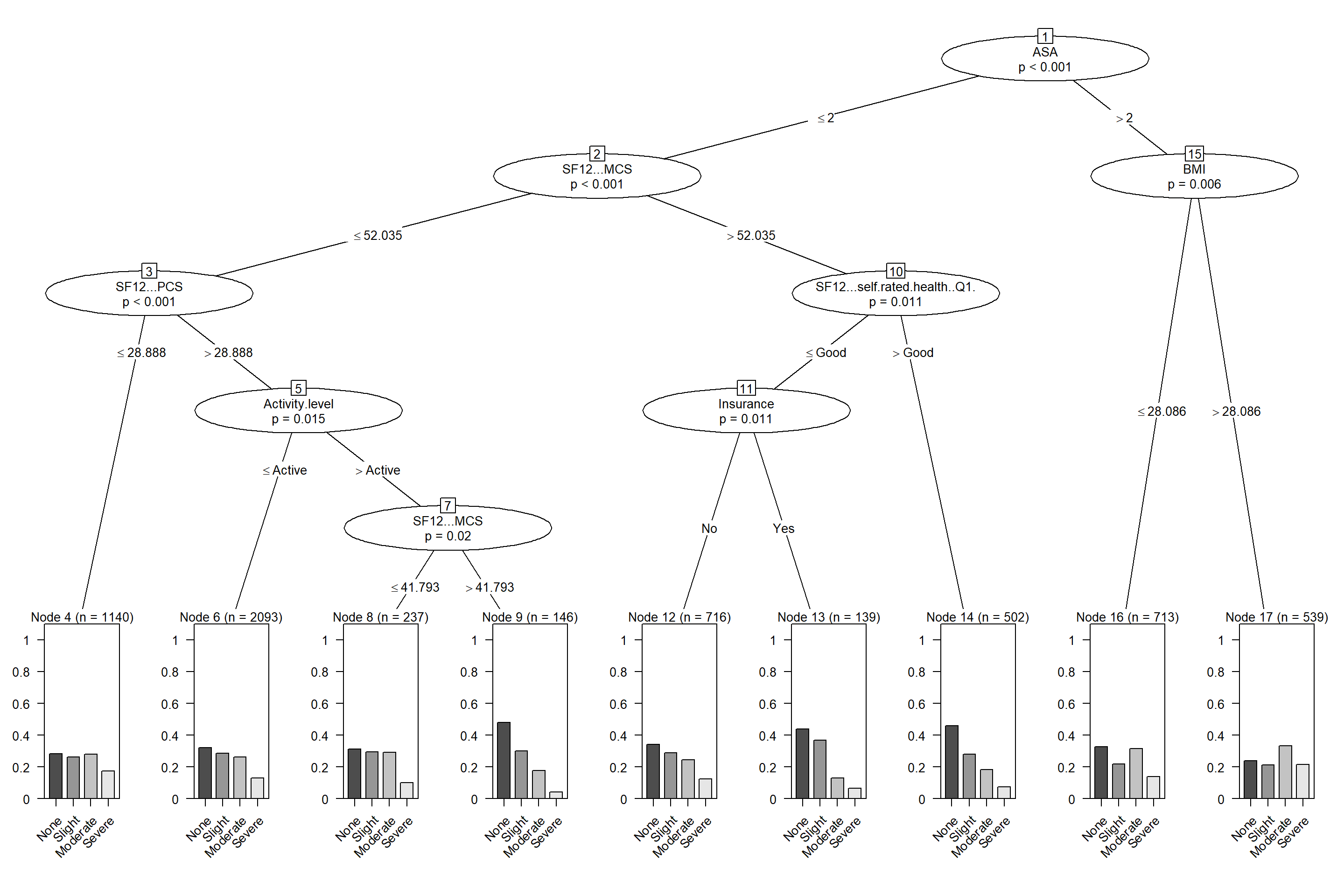


Figure S13: Ability to get in/out of a car – year 10


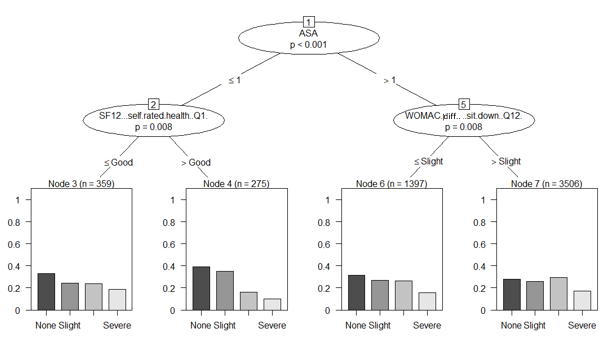


Figure S14: Dress themselves autonomously – year 1


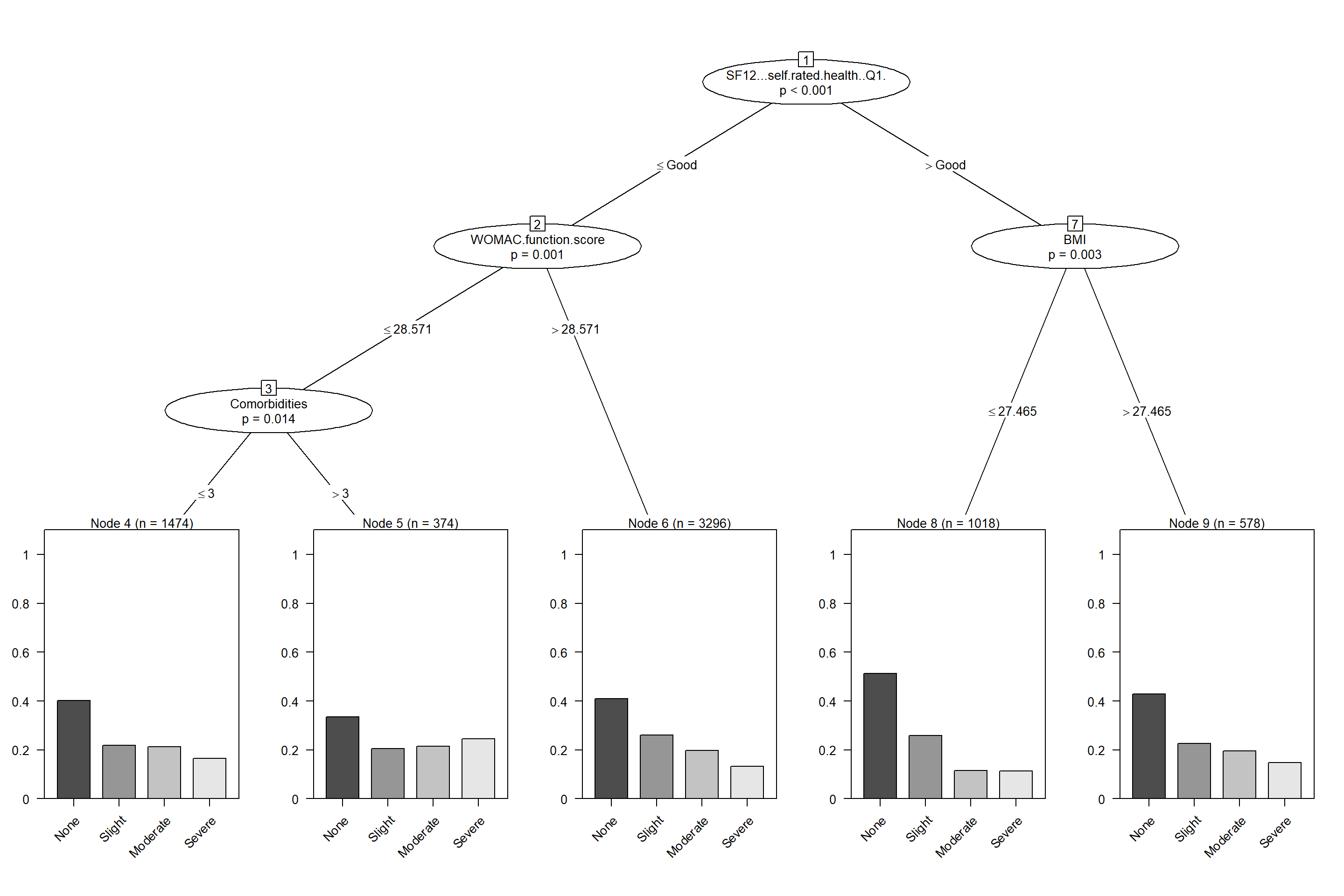


Figure S15: Dress themselves autonomously – year 5


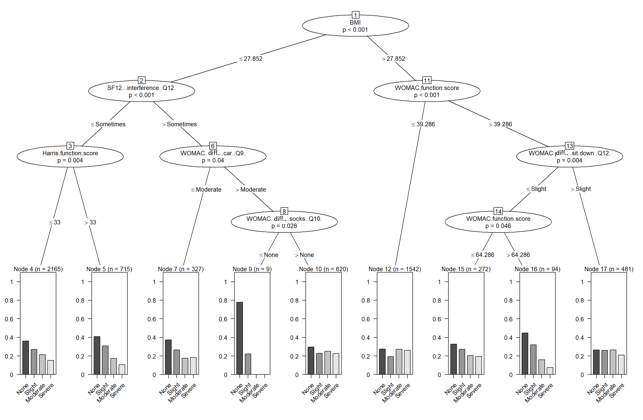


Figure S16: Dress themselves autonomously – year 10


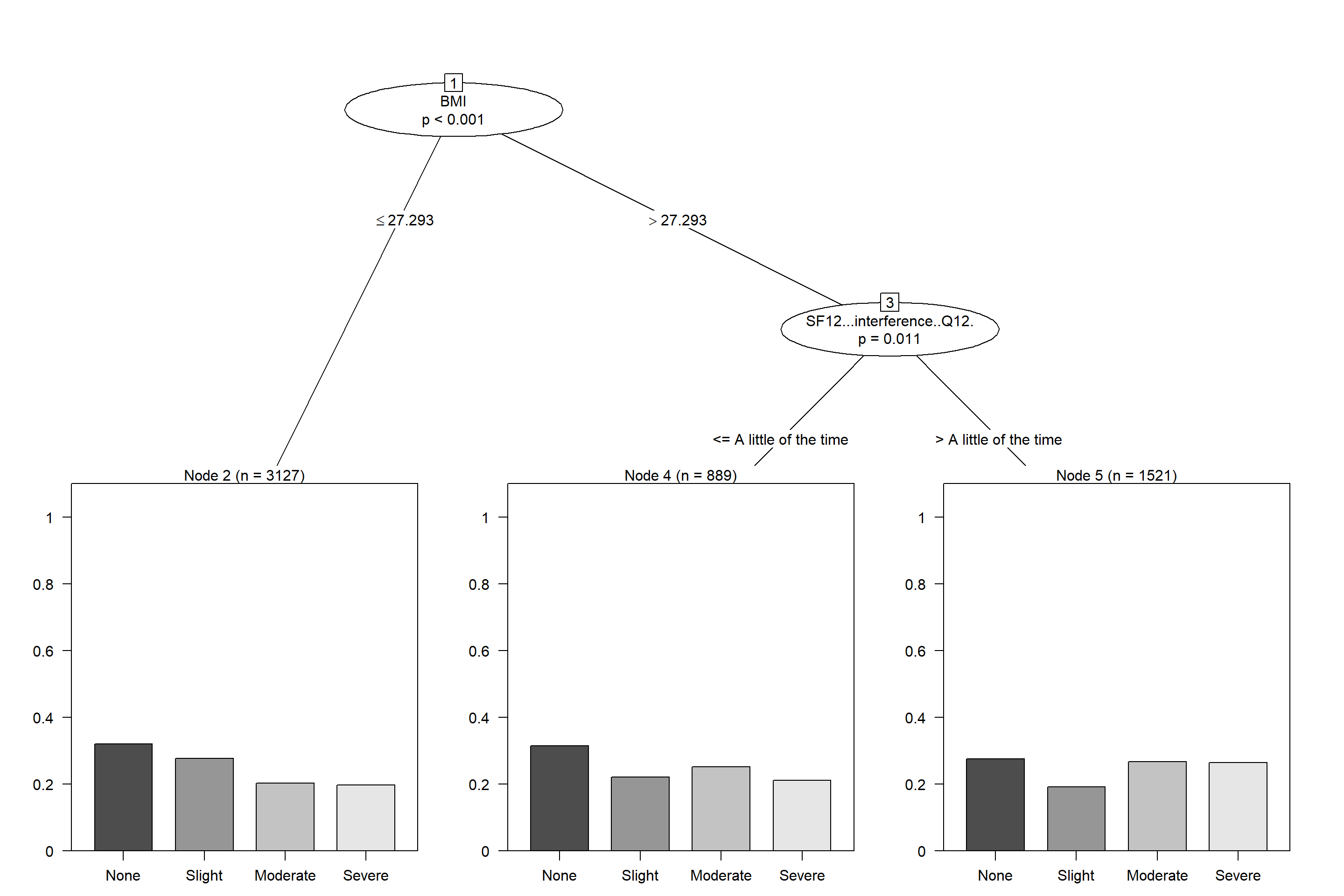


Table S2: Comparison between observed and imputed data stratified by age, SF12 question 4, Baseline

| **Age group** | **SF12 question 4 Baseline** | **Observed n** | **Observed Percentage** | **Imputed n** | **Imputed Percentage** |
| --- | --- | --- | --- | --- | --- |
| <60 | No | 102 | 13.95 | 101 | 15.71 |
| <60 | Yes | 629 | 86.05 | 542 | 84.29 |
| 60-75 | No | 296 | 15.71 | 199 | 14.54 |
| 60-75 | Yes | 1588 | 84.29 | 1170 | 85.46 |
| >75 | No | 172 | 13.76 | 130 | 13.56 |
| >75 | Yes | 1078 | 86.24 | 829 | 86.44 |

Table S3: Comparison between observed and imputed data stratified by age, SF12 question 4, Year 1

| **Age group** | **SF12 question 4 Year 1** | **Observed n** | **Observed Percentage** | **Imputed n** | **Imputed Percentage** |
| --- | --- | --- | --- | --- | --- |
| <60 | No | 260 | 63.11 | 601 | 62.73 |
| <60 | Yes | 152 | 36.89 | 357 | 37.27 |
| 60-75 | No | 590 | 66.82 | 1432 | 61.2 |
| 60-75 | Yes | 293 | 33.18 | 908 | 38.8 |
| >75 | No | 349 | 52.4 | 836 | 56.45 |
| >75 | Yes | 317 | 47.6 | 645 | 43.55 |

Table S4: Comparison between observed and imputed data stratified by age, SF12 question 4, Year 5

| **Age group** | **SF12 question 4 Year 5** | **Observed n** | **Observed Percentage** | **Imputed n** | **Imputed Percentage** |
| --- | --- | --- | --- | --- | --- |
| <60 | No | 315 | 57.17 | 455 | 57.81 |
| <60 | Yes | 236 | 42.83 | 332 | 42.19 |
| 60-75 | No | 946 | 59.95 | 804 | 54.58 |
| 60-75 | Yes | 632 | 40.05 | 669 | 45.42 |
| >75 | No | 363 | 46.07 | 481 | 45.9 |
| >75 | Yes | 425 | 53.93 | 567 | 54.1 |

Caption:

Table S5: Comparison between observed and imputed data stratified by age, SF12 question 4, Year 10

| **Age group** | **SF12 question 4 Year 10** | **Observed n** | **Observed Percentage** | **Imputed n** | **Imputed Percentage** |
| --- | --- | --- | --- | --- | --- |
| <60 | No | 151 | 52.8 | 593 | 57.97 |
| <60 | Yes | 135 | 47.2 | 430 | 42.03 |
| 60-75 | No | 466 | 56.14 | 974 | 49.07 |
| 60-75 | Yes | 364 | 43.86 | 1011 | 50.93 |
| >75 | No | 86 | 40.38 | 464 | 38.67 |
| >75 | Yes | 127 | 59.62 | 736 | 61.33 |

Table S6: Comparison between observed and imputed data stratified by sex, SF12 question 4, Baseline

| **Sex** | **SF12 question 4 Baseline** | **Observed n** | **Observed Percentage** | **Imputed n** | **Imputed Percentage** |
| --- | --- | --- | --- | --- | --- |
| F | No | 267 | 12.3 | 222 | 12.94 |
| F | Yes | 1904 | 87.7 | 1493 | 87.06 |
| M | No | 303 | 17.89 | 208 | 16.56 |
| M | Yes | 1391 | 82.11 | 1048 | 83.44 |

Table S7: Comparison between observed and imputed data stratified by sex, SF12 question 4, Year 1

| **Sex** | **SF12 question 4 Year 1** | **Observed n** | **Observed Percentage** | **Imputed n** | **Imputed Percentage** |
| --- | --- | --- | --- | --- | --- |
| F | No | 620 | 57.35 | 1579 | 57.33 |
| F | Yes | 461 | 42.65 | 1175 | 42.67 |
| M | No | 579 | 65.8 | 1290 | 63.7 |
| M | Yes | 301 | 34.2 | 735 | 36.3 |

Table S8: Comparison between observed and imputed data stratified by sex, SF12 question 4, Year 5

| **Sex** | **SF12 question 4 Year 5** | **Observed n** | **Observed Percentage** | **Imputed n** | **Imputed Percentage** |
| --- | --- | --- | --- | --- | --- |
| F | No | 875 | 52.62 | 953 | 50.05 |
| F | Yes | 788 | 47.38 | 951 | 49.95 |
| M | No | 749 | 59.73 | 787 | 56.05 |
| M | Yes | 505 | 40.27 | 617 | 43.95 |

Table S9: Comparison between observed and imputed data stratified by sex, SF12 question 4, Year 10

| **Sex** | **SF12 question 4 Year 10** | **Observed n** | **Observed Percentage** | **Imputed n** | **Imputed Percentage** |
| --- | --- | --- | --- | --- | --- |
| F | No | 397 | 52.17 | 1099 | 45.66 |
| F | Yes | 364 | 47.83 | 1308 | 54.34 |
| M | No | 306 | 53.87 | 932 | 51.75 |
| M | Yes | 262 | 46.13 | 869 | 48.25 |

Table S10: Comparison between observed and imputed data stratified by BMI, SF12 question 4, Baseline

| **BMI group** | **SF12 question 4 Baseline** | **Observed n** | **Observed Percentage** | **Imputed n** | **Imputed Percentage** |
| --- | --- | --- | --- | --- | --- |
| Healthy weight | No | 200 | 14.91 | 149 | 13.71 |
| Healthy weight | Yes | 1141 | 85.09 | 938 | 86.29 |
| Obese | No | 108 | 11.83 | 94 | 13.37 |
| Obese | Yes | 805 | 88.17 | 609 | 86.63 |
| Overweight | No | 245 | 16.45 | 179 | 16.7 |
| Overweight | Yes | 1244 | 83.55 | 893 | 83.3 |
| Severely obese | No | 3 | 5.66 | 2 | 5.13 |
| Severely obese | Yes | 50 | 94.34 | 37 | 94.87 |
| Underweight | No | 14 | 20.29 | 6 | 8.57 |
| Underweight | Yes | 55 | 79.71 | 64 | 91.43 |

Table S11: Comparison between observed and imputed data stratified by BMI, SF12 question 4, Year 1

| **BMI group** | **SF12 question 4 Year 1** | **Observed n** | **Observed Percentage** | **Imputed n** | **Imputed Percentage** |
| --- | --- | --- | --- | --- | --- |
| Healthy weight | No | 445 | 63.48 | 1061 | 62.78 |
| Healthy weight | Yes | 256 | 36.52 | 629 | 37.22 |
| Obese | No | 259 | 57.68 | 600 | 52.13 |
| Obese | Yes | 190 | 42.32 | 551 | 47.87 |
| Overweight | No | 447 | 61.15 | 1137 | 63.48 |
| Overweight | Yes | 284 | 38.85 | 654 | 36.52 |
| Severely obese | No | 17 | 50 | 21 | 37.5 |
| Severely obese | Yes | 17 | 50 | 35 | 62.5 |
| Underweight | No | 31 | 67.39 | 50 | 54.95 |
| Underweight | Yes | 15 | 32.61 | 41 | 45.05 |

Table S12: Comparison between observed and imputed data stratified by BMI, SF12 question 4, Year 5

| **BMI group** | **SF12 question 4 Year 5** | **Observed n** | **Observed Percentage** | **Imputed n** | **Imputed Percentage** |
| --- | --- | --- | --- | --- | --- |
| Healthy weight | No | 585 | 57.92 | 638 | 53.7 |
| Healthy weight | Yes | 425 | 42.08 | 550 | 46.3 |
| Obese | No | 325 | 48.29 | 380 | 46.74 |
| Obese | Yes | 348 | 51.71 | 433 | 53.26 |
| Overweight | No | 679 | 58.64 | 665 | 56.26 |
| Overweight | Yes | 479 | 41.36 | 517 | 43.74 |
| Severely obese | No | 7 | 26.92 | 18 | 30 |
| Severely obese | Yes | 19 | 73.08 | 42 | 70 |
| Underweight | No | 28 | 56 | 39 | 60 |
| Underweight | Yes | 22 | 44 | 26 | 40 |

Table S13: Comparison between observed and imputed data stratified by BMI, SF12 question 4, Year 10

| **BMI group** | **SF12 question 4 Year 10** | **Observed n** | **Observed Percentage** | **Imputed n** | **Imputed Percentage** |
| --- | --- | --- | --- | --- | --- |
| Healthy weight | No | 243 | 56.51 | 708 | 47.14 |
| Healthy weight | Yes | 187 | 43.49 | 794 | 52.86 |
| Obese | No | 158 | 48.32 | 475 | 47.36 |
| Obese | Yes | 169 | 51.68 | 528 | 52.64 |
| Overweight | No | 290 | 53.02 | 781 | 50.26 |
| Overweight | Yes | 257 | 46.98 | 773 | 49.74 |
| Severely obese | No | 4 | 40 | 30 | 43.48 |
| Severely obese | Yes | 6 | 60 | 39 | 56.52 |
| Underweight | No | 8 | 53.33 | 37 | 46.25 |
| Underweight | Yes | 7 | 46.67 | 43 | 53.75 |

Table S14: Comparison between observed and imputed data stratified by age, UCLA, Baseline

| **Age group** | **UCLA Baseline** | **Observed n** | **Observed Percentage** | **Imputed n** | **Imputed Percentage** |
| --- | --- | --- | --- | --- | --- |
| <60 | 1 | 19 | 5.07 | 65 | 6.51 |
| <60 | 2 | 85 | 22.67 | 255 | 25.53 |
| <60 | 3 | 101 | 26.93 | 269 | 26.93 |
| <60 | 4 | 74 | 19.73 | 201 | 20.12 |
| <60 | 5 | 34 | 9.07 | 62 | 6.21 |
| <60 | 6 | 33 | 8.8 | 90 | 9.01 |
| <60 | 7 | 11 | 2.93 | 31 | 3.1 |
| <60 | 8 | 3 | 0.8 | 9 | 0.9 |
| <60 | 9 | 9 | 2.4 | 15 | 1.5 |
| <60 | 10 | 6 | 1.6 | 2 | 0.2 |
| 60-75 | 1 | 39 | 4.97 | 152 | 6.16 |
| 60-75 | 2 | 200 | 25.51 | 666 | 26.97 |
| 60-75 | 3 | 223 | 28.44 | 625 | 25.31 |
| 60-75 | 4 | 161 | 20.54 | 473 | 19.16 |
| 60-75 | 5 | 65 | 8.29 | 196 | 7.94 |
| 60-75 | 6 | 61 | 7.78 | 208 | 8.42 |
| 60-75 | 7 | 19 | 2.42 | 67 | 2.71 |
| 60-75 | 8 | 7 | 0.89 | 28 | 1.13 |
| 60-75 | 9 | 9 | 1.15 | 38 | 1.54 |
| 60-75 | 10 |  |  | 16 | 0.65 |
| >75 | 1 | 59 | 10.67 | 153 | 9.24 |
| >75 | 2 | 147 | 26.58 | 538 | 32.49 |
| >75 | 3 | 146 | 26.4 | 435 | 26.27 |
| >75 | 4 | 124 | 22.42 | 272 | 16.43 |
| >75 | 5 | 30 | 5.42 | 92 | 5.56 |
| >75 | 6 | 36 | 6.51 | 104 | 6.28 |
| >75 | 7 | 8 | 1.45 | 36 | 2.17 |
| >75 | 8 | 2 | 0.36 | 8 | 0.48 |
| >75 | 9 | 1 | 0.18 | 9 | 0.54 |
| >75 | 10 |  |  | 9 | 0.54 |

Table S15: Comparison between observed and imputed data stratified by age, UCLA, Year 1

| **Age group** | **UCLA Year 1** | **Observed n** | **Observed Percentage** | **Imputed n** | **Imputed Percentage** |
| --- | --- | --- | --- | --- | --- |
| <60 | 1 | 4 | 1.07 | 8 | 0.8 |
| <60 | 2 | 21 | 5.6 | 36 | 3.62 |
| <60 | 3 | 42 | 11.2 | 126 | 12.66 |
| <60 | 4 | 86 | 22.93 | 298 | 29.95 |
| <60 | 5 | 27 | 7.2 | 66 | 6.63 |
| <60 | 6 | 61 | 16.27 | 200 | 20.1 |
| <60 | 7 | 60 | 16 | 129 | 12.96 |
| <60 | 8 | 4 | 1.07 | 18 | 1.81 |
| <60 | 9 | 32 | 8.53 | 91 | 9.15 |
| <60 | 10 | 38 | 10.13 | 23 | 2.31 |
| 60-75 | 1 | 4 | 0.51 | 37 | 1.51 |
| 60-75 | 2 | 27 | 3.47 | 121 | 4.95 |
| 60-75 | 3 | 100 | 12.85 | 370 | 15.13 |
| 60-75 | 4 | 275 | 35.35 | 813 | 33.25 |
| 60-75 | 5 | 38 | 4.88 | 124 | 5.07 |
| 60-75 | 6 | 164 | 21.08 | 453 | 18.53 |
| 60-75 | 7 | 72 | 9.25 | 244 | 9.98 |
| 60-75 | 8 | 10 | 1.29 | 39 | 1.6 |
| 60-75 | 9 | 54 | 6.94 | 156 | 6.38 |
| 60-75 | 10 | 34 | 4.37 | 88 | 3.6 |
| >75 | 1 | 11 | 1.87 | 39 | 2.5 |
| >75 | 2 | 62 | 10.54 | 121 | 7.76 |
| >75 | 3 | 138 | 23.47 | 274 | 17.58 |
| >75 | 4 | 218 | 37.07 | 516 | 33.1 |
| >75 | 5 | 30 | 5.1 | 86 | 5.52 |
| >75 | 6 | 91 | 15.48 | 249 | 15.97 |
| >75 | 7 | 20 | 3.4 | 119 | 7.63 |
| >75 | 8 | 4 | 0.68 | 13 | 0.83 |
| >75 | 9 | 10 | 1.7 | 46 | 2.95 |
| >75 | 10 | 4 | 0.68 | 96 | 6.16 |

Table S16: Comparison between observed and imputed data stratified by age, UCLA, Year 5

| **Age group** | **UCLA Year 5** | **Observed n** | **Observed Percentage** | **Imputed n** | **Imputed Percentage** |
| --- | --- | --- | --- | --- | --- |
| <60 | 2 | 5 | 1.19 | 24 | 2.62 |
| <60 | 3 | 20 | 4.75 | 29 | 3.16 |
| <60 | 4 | 36 | 8.55 | 75 | 8.18 |
| <60 | 5 | 65 | 15.44 | 119 | 12.98 |
| <60 | 6 | 108 | 25.65 | 291 | 31.73 |
| <60 | 7 | 63 | 14.96 | 154 | 16.79 |
| <60 | 8 | 44 | 10.45 | 124 | 13.52 |
| <60 | 9 | 45 | 10.69 | 93 | 10.14 |
| <60 | 10 | 35 | 8.31 | 7 | 0.76 |
| <60 | 1 |  |  | 1 | 0.11 |
| 60-75 | 1 | 6 | 0.54 | 29 | 1.5 |
| 60-75 | 2 | 28 | 2.51 | 74 | 3.83 |
| 60-75 | 3 | 46 | 4.12 | 99 | 5.12 |
| 60-75 | 4 | 158 | 14.15 | 291 | 15.05 |
| 60-75 | 5 | 194 | 17.37 | 344 | 17.79 |
| 60-75 | 6 | 344 | 30.8 | 575 | 29.73 |
| 60-75 | 7 | 164 | 14.68 | 253 | 13.08 |
| 60-75 | 8 | 87 | 7.79 | 131 | 6.77 |
| 60-75 | 9 | 47 | 4.21 | 67 | 3.46 |
| 60-75 | 10 | 43 | 3.85 | 71 | 3.67 |
| >75 | 1 | 12 | 2.4 | 35 | 2.62 |
| >75 | 2 | 58 | 11.6 | 96 | 7.19 |
| >75 | 3 | 67 | 13.4 | 143 | 10.7 |
| >75 | 4 | 112 | 22.4 | 260 | 19.46 |
| >75 | 5 | 95 | 19 | 250 | 18.71 |
| >75 | 6 | 114 | 22.8 | 299 | 22.38 |
| >75 | 7 | 20 | 4 | 90 | 6.74 |
| >75 | 8 | 17 | 3.4 | 45 | 3.37 |
| >75 | 9 | 3 | 0.6 | 16 | 1.2 |
| >75 | 10 | 2 | 0.4 | 102 | 7.63 |

Table S17: Comparison between observed and imputed data stratified by age, UCLA, Year 10

| **Age group** | **UCLA Year 10** | **Observed n** | **Observed Percentage** | **Imputed n** | **Imputed Percentage** |
| --- | --- | --- | --- | --- | --- |
| <60 | 2 | 9 | 3.32 | 28 | 2.7 |
| <60 | 3 | 14 | 5.17 | 43 | 4.14 |
| <60 | 4 | 23 | 8.49 | 87 | 8.38 |
| <60 | 5 | 42 | 15.5 | 153 | 14.74 |
| <60 | 6 | 68 | 25.09 | 278 | 26.78 |
| <60 | 7 | 46 | 16.97 | 187 | 18.02 |
| <60 | 8 | 41 | 15.13 | 136 | 13.1 |
| <60 | 9 | 22 | 8.12 | 108 | 10.4 |
| <60 | 10 | 6 | 2.21 | 11 | 1.06 |
| <60 | 1 |  |  | 7 | 0.67 |
| 60-75 | 1 | 16 | 2.17 | 67 | 3.23 |
| 60-75 | 2 | 37 | 5.01 | 129 | 6.21 |
| 60-75 | 3 | 61 | 8.27 | 217 | 10.45 |
| 60-75 | 4 | 109 | 14.77 | 340 | 16.37 |
| 60-75 | 5 | 150 | 20.33 | 427 | 20.56 |
| 60-75 | 6 | 205 | 27.78 | 476 | 22.92 |
| 60-75 | 7 | 79 | 10.7 | 212 | 10.21 |
| 60-75 | 8 | 36 | 4.88 | 106 | 5.1 |
| 60-75 | 9 | 26 | 3.52 | 60 | 2.89 |
| 60-75 | 10 | 19 | 2.57 | 43 | 2.07 |
| >75 | 1 | 13 | 6.6 | 100 | 8.22 |
| >75 | 2 | 36 | 18.27 | 179 | 14.72 |
| >75 | 3 | 29 | 14.72 | 188 | 15.46 |
| >75 | 4 | 50 | 25.38 | 230 | 18.91 |
| >75 | 5 | 32 | 16.24 | 217 | 17.85 |
| >75 | 6 | 26 | 13.2 | 161 | 13.24 |
| >75 | 7 | 5 | 2.54 | 31 | 2.55 |
| >75 | 8 | 4 | 2.03 | 25 | 2.06 |
| >75 | 9 | 2 | 1.02 | 16 | 1.32 |
| >75 | 10 |  |  | 69 | 5.67 |

Table S18: Comparison between observed and imputed data stratified by sex, UCLA, Baseline

| **Sex** | **UCLA Baseline** | **Observed n** | **Observed Percentage** | **Imputed n** | **Imputed Percentage** |
| --- | --- | --- | --- | --- | --- |
| F | 1 | 70 | 7.46 | 245 | 8.31 |
| F | 2 | 231 | 24.63 | 863 | 29.27 |
| F | 3 | 276 | 29.42 | 773 | 26.22 |
| F | 4 | 199 | 21.22 | 534 | 18.11 |
| F | 5 | 77 | 8.21 | 189 | 6.41 |
| F | 6 | 63 | 6.72 | 200 | 6.78 |
| F | 7 | 14 | 1.49 | 72 | 2.44 |
| F | 8 | 1 | 0.11 | 21 | 0.71 |
| F | 9 | 6 | 0.64 | 34 | 1.15 |
| F | 10 | 1 | 0.11 | 17 | 0.58 |
| M | 1 | 47 | 6.07 | 125 | 5.74 |
| M | 2 | 201 | 25.97 | 596 | 27.39 |
| M | 3 | 194 | 25.06 | 556 | 25.55 |
| M | 4 | 160 | 20.67 | 412 | 18.93 |
| M | 5 | 52 | 6.72 | 161 | 7.4 |
| M | 6 | 67 | 8.66 | 202 | 9.28 |
| M | 7 | 24 | 3.1 | 62 | 2.85 |
| M | 8 | 11 | 1.42 | 24 | 1.1 |
| M | 9 | 13 | 1.68 | 28 | 1.29 |
| M | 10 | 5 | 0.65 | 10 | 0.46 |

Table S19: Comparison between observed and imputed data stratified by sex, UCLA, Year 1

| **Sex** | **UCLA Year 1** | **Observed n** | **Observed Percentage** | **Imputed n** | **Imputed Percentage** |
| --- | --- | --- | --- | --- | --- |
| F | 1 | 9 | 0.94 | 52 | 1.81 |
| F | 2 | 52 | 5.41 | 174 | 6.05 |
| F | 3 | 164 | 17.07 | 444 | 15.45 |
| F | 4 | 357 | 37.15 | 966 | 33.61 |
| F | 5 | 48 | 4.99 | 136 | 4.73 |
| F | 6 | 205 | 21.33 | 517 | 17.99 |
| F | 7 | 52 | 5.41 | 263 | 9.15 |
| F | 8 | 5 | 0.52 | 32 | 1.11 |
| F | 9 | 35 | 3.64 | 161 | 5.6 |
| F | 10 | 34 | 3.54 | 129 | 4.49 |
| M | 1 | 10 | 1.28 | 32 | 1.51 |
| M | 2 | 58 | 7.44 | 104 | 4.89 |
| M | 3 | 116 | 14.87 | 326 | 15.34 |
| M | 4 | 222 | 28.46 | 661 | 31.11 |
| M | 5 | 47 | 6.03 | 140 | 6.59 |
| M | 6 | 111 | 14.23 | 385 | 18.12 |
| M | 7 | 100 | 12.82 | 229 | 10.78 |
| M | 8 | 13 | 1.67 | 38 | 1.79 |
| M | 9 | 61 | 7.82 | 132 | 6.21 |
| M | 10 | 42 | 5.38 | 78 | 3.67 |

Table S20: Comparison between observed and imputed data stratified by sex, UCLA, Year 5

| **Sex** | **UCLA Year 5** | **Observed n** | **Observed Percentage** | **Imputed n** | **Imputed Percentage** |
| --- | --- | --- | --- | --- | --- |
| F | 1 | 16 | 1.37 | 51 | 2.13 |
| F | 2 | 65 | 5.56 | 140 | 5.84 |
| F | 3 | 85 | 7.26 | 188 | 7.84 |
| F | 4 | 196 | 16.75 | 427 | 17.81 |
| F | 5 | 225 | 19.23 | 406 | 16.94 |
| F | 6 | 341 | 29.15 | 591 | 24.66 |
| F | 7 | 107 | 9.15 | 245 | 10.22 |
| F | 8 | 76 | 6.5 | 146 | 6.09 |
| F | 9 | 32 | 2.74 | 71 | 2.96 |
| F | 10 | 27 | 2.31 | 132 | 5.51 |
| M | 1 | 2 | 0.23 | 14 | 0.78 |
| M | 2 | 26 | 3 | 54 | 3.02 |
| M | 3 | 48 | 5.53 | 83 | 4.64 |
| M | 4 | 110 | 12.67 | 199 | 11.12 |
| M | 5 | 129 | 14.86 | 307 | 17.15 |
| M | 6 | 225 | 25.92 | 574 | 32.07 |
| M | 7 | 140 | 16.13 | 252 | 14.08 |
| M | 8 | 72 | 8.29 | 154 | 8.6 |
| M | 9 | 63 | 7.26 | 105 | 5.87 |
| M | 10 | 53 | 6.11 | 48 | 2.68 |

Table S21: Comparison between observed and imputed data stratified by sex, UCLA, Year 10

| **Sex** | **UCLA Year 10** | **Observed n** | **Observed Percentage** | **Imputed n** | **Imputed Percentage** |
| --- | --- | --- | --- | --- | --- |
| F | 1 | 22 | 3.24 | 134 | 5.38 |
| F | 2 | 62 | 9.13 | 239 | 9.6 |
| F | 3 | 63 | 9.28 | 287 | 11.53 |
| F | 4 | 126 | 18.56 | 425 | 17.08 |
| F | 5 | 118 | 17.38 | 466 | 18.72 |
| F | 6 | 175 | 25.77 | 469 | 18.84 |
| F | 7 | 62 | 9.13 | 191 | 7.67 |
| F | 8 | 31 | 4.57 | 104 | 4.18 |
| F | 9 | 13 | 1.91 | 74 | 2.97 |
| F | 10 | 7 | 1.03 | 100 | 4.02 |
| M | 1 | 7 | 1.33 | 40 | 2.17 |
| M | 2 | 20 | 3.8 | 97 | 5.27 |
| M | 3 | 41 | 7.78 | 161 | 8.74 |
| M | 4 | 56 | 10.63 | 232 | 12.6 |
| M | 5 | 106 | 20.11 | 331 | 17.97 |
| M | 6 | 124 | 23.53 | 446 | 24.21 |
| M | 7 | 68 | 12.9 | 239 | 12.98 |
| M | 8 | 50 | 9.49 | 163 | 8.85 |
| M | 9 | 37 | 7.02 | 110 | 5.97 |
| M | 10 | 18 | 3.42 | 23 | 1.25 |

Table S22: Comparison between observed and imputed data stratified by BMI, UCLA, Baseline

| **BMI group** | **UCLA Baseline** | **Observed n** | **Observed Percentage** | **Imputed n** | **Imputed Percentage** |
| --- | --- | --- | --- | --- | --- |
| Healthy weight | 1 | 40 | 6.77 | 110 | 5.99 |
| Healthy weight | 2 | 129 | 21.83 | 468 | 25.48 |
| Healthy weight | 3 | 168 | 28.43 | 487 | 26.51 |
| Healthy weight | 4 | 134 | 22.67 | 356 | 19.38 |
| Healthy weight | 5 | 51 | 8.63 | 130 | 7.08 |
| Healthy weight | 6 | 37 | 6.26 | 168 | 9.15 |
| Healthy weight | 7 | 19 | 3.21 | 53 | 2.89 |
| Healthy weight | 8 | 4 | 0.68 | 18 | 0.98 |
| Healthy weight | 9 | 6 | 1.02 | 35 | 1.91 |
| Healthy weight | 10 | 3 | 0.51 | 12 | 0.65 |
| Obese | 1 | 29 | 7.16 | 116 | 9.58 |
| Obese | 2 | 133 | 32.84 | 416 | 34.35 |
| Obese | 3 | 110 | 27.16 | 315 | 26.01 |
| Obese | 4 | 82 | 20.25 | 186 | 15.36 |
| Obese | 5 | 22 | 5.43 | 66 | 5.45 |
| Obese | 6 | 22 | 5.43 | 67 | 5.53 |
| Obese | 7 | 2 | 0.49 | 23 | 1.9 |
| Obese | 8 | 2 | 0.49 | 11 | 0.91 |
| Obese | 9 | 2 | 0.49 | 6 | 0.5 |
| Obese | 10 | 1 | 0.25 | 5 | 0.41 |
| Overweight | 1 | 43 | 6.64 | 126 | 6.59 |
| Overweight | 2 | 154 | 23.77 | 517 | 27.03 |
| Overweight | 3 | 174 | 26.85 | 491 | 25.67 |
| Overweight | 4 | 127 | 19.6 | 377 | 19.71 |
| Overweight | 5 | 47 | 7.25 | 145 | 7.58 |
| Overweight | 6 | 68 | 10.49 | 158 | 8.26 |
| Overweight | 7 | 16 | 2.47 | 57 | 2.98 |
| Overweight | 8 | 6 | 0.93 | 14 | 0.73 |
| Overweight | 9 | 11 | 1.7 | 20 | 1.05 |
| Overweight | 10 | 2 | 0.31 | 8 | 0.42 |
| Severely obese | 1 | 5 | 17.24 | 10 | 15.87 |
| Severely obese | 2 | 9 | 31.03 | 27 | 42.86 |
| Severely obese | 3 | 7 | 24.14 | 14 | 22.22 |
| Severely obese | 4 | 4 | 13.79 | 6 | 9.52 |
| Severely obese | 5 | 1 | 3.45 | 4 | 6.35 |
| Severely obese | 6 | 2 | 6.9 |  |  |
| Severely obese | 7 | 1 | 3.45 |  |  |
| Severely obese | 10 |  |  | 2 | 3.17 |
| Underweight | 2 | 7 | 17.95 | 31 | 31 |
| Underweight | 3 | 11 | 28.21 | 22 | 22 |
| Underweight | 4 | 12 | 30.77 | 21 | 21 |
| Underweight | 5 | 8 | 20.51 | 5 | 5 |
| Underweight | 6 | 1 | 2.56 | 9 | 9 |
| Underweight | 1 |  |  | 8 | 8 |
| Underweight | 7 |  |  | 1 | 1 |
| Underweight | 8 |  |  | 2 | 2 |
| Underweight | 9 |  |  | 1 | 1 |

Table S23: Comparison between observed and imputed data stratified by BMI, UCLA, Year 1

| **BMI group** | **UCLA Year 1** | **Observed n** | **Observed Percentage** | **Imputed n** | **Imputed Percentage** |
| --- | --- | --- | --- | --- | --- |
| Healthy weight | 1 | 7 | 1.1 | 18 | 1.03 |
| Healthy weight | 2 | 30 | 4.69 | 91 | 5.19 |
| Healthy weight | 3 | 96 | 15.02 | 236 | 13.47 |
| Healthy weight | 4 | 207 | 32.39 | 581 | 33.16 |
| Healthy weight | 5 | 25 | 3.91 | 79 | 4.51 |
| Healthy weight | 6 | 128 | 20.03 | 330 | 18.84 |
| Healthy weight | 7 | 62 | 9.7 | 195 | 11.13 |
| Healthy weight | 8 | 5 | 0.78 | 29 | 1.66 |
| Healthy weight | 9 | 44 | 6.89 | 120 | 6.85 |
| Healthy weight | 10 | 35 | 5.48 | 73 | 4.17 |
| Obese | 1 | 3 | 0.77 | 32 | 2.65 |
| Obese | 2 | 36 | 9.18 | 72 | 5.96 |
| Obese | 3 | 71 | 18.11 | 226 | 18.71 |
| Obese | 4 | 159 | 40.56 | 415 | 34.35 |
| Obese | 5 | 25 | 6.38 | 59 | 4.88 |
| Obese | 6 | 65 | 16.58 | 195 | 16.14 |
| Obese | 7 | 22 | 5.61 | 94 | 7.78 |
| Obese | 9 | 9 | 2.3 | 43 | 3.56 |
| Obese | 10 | 2 | 0.51 | 61 | 5.05 |
| Obese | 8 |  |  | 11 | 0.91 |
| Overweight | 1 | 8 | 1.26 | 30 | 1.59 |
| Overweight | 2 | 36 | 5.68 | 107 | 5.67 |
| Overweight | 3 | 104 | 16.4 | 282 | 14.94 |
| Overweight | 4 | 191 | 30.13 | 578 | 30.61 |
| Overweight | 5 | 37 | 5.84 | 134 | 7.1 |
| Overweight | 6 | 107 | 16.88 | 354 | 18.75 |
| Overweight | 7 | 61 | 9.62 | 190 | 10.06 |
| Overweight | 8 | 13 | 2.05 | 29 | 1.54 |
| Overweight | 9 | 39 | 6.15 | 120 | 6.36 |
| Overweight | 10 | 38 | 5.99 | 64 | 3.39 |
| Severely obese | 1 | 1 | 3.03 | 3 | 5.26 |
| Severely obese | 2 | 6 | 18.18 | 4 | 7.02 |
| Severely obese | 3 | 7 | 21.21 | 6 | 10.53 |
| Severely obese | 4 | 8 | 24.24 | 22 | 38.6 |
| Severely obese | 5 | 4 | 12.12 | 1 | 1.75 |
| Severely obese | 6 | 4 | 12.12 | 11 | 19.3 |
| Severely obese | 7 | 3 | 9.09 | 4 | 7.02 |
| Severely obese | 9 |  |  | 1 | 1.75 |
| Severely obese | 10 |  |  | 5 | 8.77 |
| Underweight | 2 | 2 | 4.65 | 4 | 4.26 |
| Underweight | 3 | 2 | 4.65 | 20 | 21.28 |
| Underweight | 4 | 14 | 32.56 | 31 | 32.98 |
| Underweight | 5 | 4 | 9.3 | 3 | 3.19 |
| Underweight | 6 | 12 | 27.91 | 12 | 12.77 |
| Underweight | 7 | 4 | 9.3 | 9 | 9.57 |
| Underweight | 9 | 4 | 9.3 | 9 | 9.57 |
| Underweight | 10 | 1 | 2.33 | 4 | 4.26 |
| Underweight | 1 |  |  | 1 | 1.06 |
| Underweight | 8 |  |  | 1 | 1.06 |

Table S24: Comparison between observed and imputed data stratified by BMI, UCLA, Year 5

| **BMI group** | **UCLA Year 5** | **Observed n** | **Observed Percentage** | **Imputed n** | **Imputed Percentage** |
| --- | --- | --- | --- | --- | --- |
| Healthy weight | 1 | 6 | 0.87 | 19 | 1.26 |
| Healthy weight | 2 | 33 | 4.76 | 66 | 4.39 |
| Healthy weight | 3 | 31 | 4.47 | 88 | 5.85 |
| Healthy weight | 4 | 78 | 11.26 | 199 | 13.22 |
| Healthy weight | 5 | 115 | 16.59 | 245 | 16.28 |
| Healthy weight | 6 | 196 | 28.28 | 426 | 28.31 |
| Healthy weight | 7 | 90 | 12.99 | 210 | 13.95 |
| Healthy weight | 8 | 67 | 9.67 | 119 | 7.91 |
| Healthy weight | 9 | 41 | 5.92 | 78 | 5.18 |
| Healthy weight | 10 | 36 | 5.19 | 55 | 3.65 |
| Obese | 1 | 6 | 1.23 | 25 | 2.51 |
| Obese | 2 | 19 | 3.89 | 47 | 4.71 |
| Obese | 3 | 47 | 9.63 | 84 | 8.42 |
| Obese | 4 | 103 | 21.11 | 189 | 18.94 |
| Obese | 5 | 109 | 22.34 | 176 | 17.64 |
| Obese | 6 | 116 | 23.77 | 260 | 26.05 |
| Obese | 7 | 48 | 9.84 | 91 | 9.12 |
| Obese | 8 | 14 | 2.87 | 48 | 4.81 |
| Obese | 9 | 13 | 2.66 | 31 | 3.11 |
| Obese | 10 | 13 | 2.66 | 47 | 4.71 |
| Overweight | 1 | 5 | 0.62 | 19 | 1.24 |
| Overweight | 2 | 33 | 4.11 | 73 | 4.75 |
| Overweight | 3 | 50 | 6.23 | 88 | 5.73 |
| Overweight | 4 | 112 | 13.95 | 215 | 13.99 |
| Overweight | 5 | 119 | 14.82 | 261 | 16.98 |
| Overweight | 6 | 248 | 30.88 | 439 | 28.56 |
| Overweight | 7 | 107 | 13.33 | 184 | 11.97 |
| Overweight | 8 | 64 | 7.97 | 123 | 8 |
| Overweight | 9 | 36 | 4.48 | 64 | 4.16 |
| Overweight | 10 | 29 | 3.61 | 71 | 4.62 |
| Severely obese | 1 | 1 | 3.57 | 1 | 1.72 |
| Severely obese | 2 | 2 | 7.14 | 4 | 6.9 |
| Severely obese | 3 | 4 | 14.29 | 8 | 13.79 |
| Severely obese | 4 | 11 | 39.29 | 10 | 17.24 |
| Severely obese | 5 | 7 | 25 | 14 | 24.14 |
| Severely obese | 8 | 2 | 7.14 | 1 | 1.72 |
| Severely obese | 10 | 1 | 3.57 | 5 | 8.62 |
| Severely obese | 6 |  |  | 11 | 18.97 |
| Severely obese | 7 |  |  | 2 | 3.45 |
| Severely obese | 9 |  |  | 2 | 3.45 |
| Underweight | 2 | 4 | 15.38 | 4 | 4.49 |
| Underweight | 3 | 1 | 3.85 | 3 | 3.37 |
| Underweight | 4 | 2 | 7.69 | 13 | 14.61 |
| Underweight | 5 | 4 | 15.38 | 17 | 19.1 |
| Underweight | 6 | 6 | 23.08 | 29 | 32.58 |
| Underweight | 7 | 2 | 7.69 | 10 | 11.24 |
| Underweight | 8 | 1 | 3.85 | 9 | 10.11 |
| Underweight | 9 | 5 | 19.23 | 1 | 1.12 |
| Underweight | 10 | 1 | 3.85 | 2 | 2.25 |
| Underweight | 1 |  |  | 1 | 1.12 |

Table S25 Comparison between observed and imputed data stratified by BMI, UCLA, Year 10

| **BMI group** | **UCLA Year 10** | **Observed n** | **Observed Percentage** | **Imputed n** | **Imputed Percentage** |
| --- | --- | --- | --- | --- | --- |
| Healthy weight | 1 | 10 | 2.5 | 64 | 4.18 |
| Healthy weight | 2 | 28 | 7 | 113 | 7.38 |
| Healthy weight | 3 | 29 | 7.25 | 148 | 9.66 |
| Healthy weight | 4 | 58 | 14.5 | 234 | 15.27 |
| Healthy weight | 5 | 64 | 16 | 265 | 17.3 |
| Healthy weight | 6 | 85 | 21.25 | 327 | 21.34 |
| Healthy weight | 7 | 59 | 14.75 | 166 | 10.84 |
| Healthy weight | 8 | 34 | 8.5 | 101 | 6.59 |
| Healthy weight | 9 | 19 | 4.75 | 82 | 5.35 |
| Healthy weight | 10 | 14 | 3.5 | 32 | 2.09 |
| Obese | 1 | 9 | 3.05 | 51 | 4.93 |
| Obese | 2 | 24 | 8.14 | 91 | 8.79 |
| Obese | 3 | 24 | 8.14 | 111 | 10.72 |
| Obese | 4 | 51 | 17.29 | 159 | 15.36 |
| Obese | 5 | 60 | 20.34 | 209 | 20.19 |
| Obese | 6 | 83 | 28.14 | 214 | 20.68 |
| Obese | 7 | 19 | 6.44 | 83 | 8.02 |
| Obese | 8 | 16 | 5.42 | 51 | 4.93 |
| Obese | 9 | 9 | 3.05 | 27 | 2.61 |
| Obese | 10 |  |  | 39 | 3.77 |
| Overweight | 1 | 9 | 1.84 | 53 | 3.29 |
| Overweight | 2 | 25 | 5.1 | 118 | 7.32 |
| Overweight | 3 | 48 | 9.8 | 167 | 10.37 |
| Overweight | 4 | 71 | 14.49 | 237 | 14.71 |
| Overweight | 5 | 97 | 19.8 | 292 | 18.13 |
| Overweight | 6 | 127 | 25.92 | 354 | 21.97 |
| Overweight | 7 | 50 | 10.2 | 169 | 10.49 |
| Overweight | 8 | 31 | 6.33 | 105 | 6.52 |
| Overweight | 9 | 21 | 4.29 | 70 | 4.35 |
| Overweight | 10 | 11 | 2.24 | 46 | 2.86 |
| Severely obese | 2 | 3 | 37.5 | 9 | 12.68 |
| Severely obese | 3 | 2 | 25 | 10 | 14.08 |
| Severely obese | 4 | 1 | 12.5 | 14 | 19.72 |
| Severely obese | 5 | 2 | 25 | 12 | 16.9 |
| Severely obese | 1 |  |  | 4 | 5.63 |
| Severely obese | 6 |  |  | 9 | 12.68 |
| Severely obese | 7 |  |  | 7 | 9.86 |
| Severely obese | 8 |  |  | 1 | 1.41 |
| Severely obese | 9 |  |  | 2 | 2.82 |
| Severely obese | 10 |  |  | 3 | 4.23 |
| Underweight | 1 | 1 | 7.69 | 2 | 2.44 |
| Underweight | 2 | 2 | 15.38 | 5 | 6.1 |
| Underweight | 3 | 1 | 7.69 | 12 | 14.63 |
| Underweight | 4 | 1 | 7.69 | 13 | 15.85 |
| Underweight | 5 | 1 | 7.69 | 19 | 23.17 |
| Underweight | 6 | 4 | 30.77 | 11 | 13.41 |
| Underweight | 7 | 2 | 15.38 | 5 | 6.1 |
| Underweight | 9 | 1 | 7.69 | 3 | 3.66 |
| Underweight | 8 |  |  | 9 | 10.98 |
| Underweight | 10 |  |  | 3 | 3.66 |
